# Human γδ T Cell Function Is Impaired Upon Mevalonate Pathway Inhibition

**DOI:** 10.1111/imm.13931

**Published:** 2025-04-22

**Authors:** Tsz Kin Suen, Burcu Al, Thomas Ulas, Nico Reusch, Harsh Bahrar, Siroon Bekkering, Jaydeep Bhat, Dieter Kabelitz, Joachim L. Schultze, Frank L. van de Veerdonk, Jeanine Roeters van Lennep, Niels P. Riksen, Leo A. B. Joosten, Mihai G. Netea, Katarzyna Placek

**Affiliations:** ^1^ Immunology and Metabolism, Life and Medical Sciences Institute University of Bonn Bonn Germany; ^2^ Systems Medicine, German Center for Neurodegenerative Diseases (DZNE) Bonn Germany; ^3^ PRECISE Platform for Single Cell Genomics and Epigenomics at the DZNE and the University of Bonn Bonn Germany; ^4^ Genomics and Immunoregulation, Life and Medical Sciences (LIMES) Institute University of Bonn Bonn Germany; ^5^ Department of Internal Medicine Radboud University Medical Center Nijmegen the Netherlands; ^6^ Institute of Immunology University of Kiel, University Hospital Schleswig‐Holstein Campus Kiel Kiel Germany; ^7^ Department of Internal Medicine Erasmus MC University Center Rotterdam the Netherlands; ^8^ Department of Medical Genetics Iuliu Haţieganu University of Medicine and Pharmacy Cluj‐Napoca Romania

**Keywords:** cytokines, flow cytometry, human, protein kinases/phophatases, T cell

## Abstract

Vδ2 T cells, a predominant human peripheral γδ T cell population, are a promising candidate for the development of immunotherapies against cancer and infected cells. Aminobisphosphonate drugs, such as zoledronate, are commonly used to expand Vδ2 T cells. Yet, such in vitro generated cells have limited efficacy in the clinic. We found that despite inducing excessive proliferation of Vδ2 T cells, zoledronate impaired their effector function and caused the upregulation of the inhibitory receptor TIM3. This effect was due to the inhibition of mevalonate metabolism and dysregulation of downstream biological processes such as protein prenylation and intracellular signalling. In vitro and in vivo inhibition of mevalonate metabolism with zoledronate, statins, and 6‐fluoromevalonate, as well as genetic deficiency of the mevalonate kinase, all resulted in compromised cytokine and cytotoxic molecule production by Vδ2 T cells. Impaired Vδ2 T cell function was accompanied by transcriptome and kinome changes. Our findings reveal the importance of mevalonate metabolism for the proper functioning of Vδ2 T cells. This observation provides important considerations for improving their therapeutic use and has repercussions for patients with statin or aminobisphosphonate treatments.

## Graphical abstract

Mevalonate pathway is important for the effector function of gammadelta T cells. Inhibition of the pathway in vivo and in vitro impairs cytokine and cytotoxic molecule production by mainly affecting protein prenylation and disturbing intracellular signalling in Vdelta2 T cells. Created with Biorender.

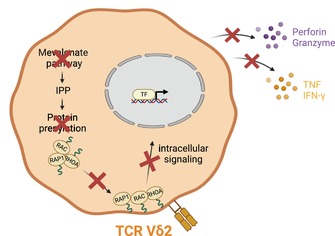

## 1 Introduction

V delta 2 (Vδ2) T cells are the predominant population of unconventional γδ T cells in human peripheral blood. The T cell receptor (TCR) of Vδ2 T cells is composed of Vδ2 and Vγ9 chains and recognises non‐peptide molecules such as isopentenyl pyrophosphate IPP [1, 2] and microbe‐derived (E)‐4‐hydroxy‐3‐methyl‐but‐2‐enyl pyrophosphate (HMBPP) in the context of butyrophilins in an MHC molecule unrestricted manner [3, 4, 5]. Upon activation, Vδ2 T cells produce pro‐inflammatory cytokines, mainly TNF and IFN‐γ [6]. They also exert cytotoxic or phagocytotic activity against infected and tumour cells [7, 8]. Due to their effective anti‐cancer activity, easy in vitro expansion with phosphoantigens and aminobisphosphonates, and suitability for allogeneic transfer, they sparked interest in cancer immunotherapy [9]. Aminobisphosphonates, such as zoledronate, are commonly used to expand Vδ2 T cells in vitro in peripheral blood mononuclear cell (PBMC) cultures. Mechanistically, they inhibit farnesyl pyrophosphate (FPP) synthase in the mevalonate pathway [10], resulting in the accumulation of its substrate IPP. Elevated levels of IPP lead to activation of the butyrophilin complex on antigen‐presenting cells [10, 11, 12], which is then recognised by the Vδ2Vγ9 TCR and activates Vδ2Vγ9 T cells (further referred to as Vδ2), resulting in their proliferation [13, 14]. Furthermore, recent reports show that zoledronate also causes the upregulation of butyrophilin 3A (BTN3A) expression in Daudi lymphoma cells, accounting for the efficient killing of the cells by Vδ2 T cells [15]. Yet, the effectiveness of zoledronate‐expanded Vδ2 T cells in cancer patients remains limited [16, 17]. While zoledronate has been proven to be an efficient Vδ2 T cell proliferation‐inducing agent, its inhibitory effect on mevalonate metabolism in the Vδ2 T cells and their immune function is poorly defined.

Indeed, the mevalonate pathway is an important metabolic process that leads to the synthesis of isoprenoids and sterols in eukaryotes, archaea, and some bacteria and is the initial step for many biological processes [18, 19]. The entry substrate for the pathway, acetyl‐CoA, is transformed into several intermediate metabolites such as β‐hydroxy β‐methylglutaryl‐coenzyme A (HMG‐CoA), mevalonate, the aforementioned IPP and FPP. FPP can then be used for cholesterol synthesis, the production of dolichol, or protein prenylation: geranylgeranylation and farnesylation [20]. Cholesterol is an important lipid component of cellular membranes, where it modulates their fluidity and permeability [21]. Being a component of lipid rafts, it is also involved in receptor signalling [22]. Dolichol is a lipid carrier for the protein N‐glycosylation, a post‐translational modification that changes the nature of target proteins [23] by affecting protein localisation, stability, interaction with other proteins and function [24, 25, 26]. Furthermore, protein prenylation increases protein anchoring to cellular membranes and therefore affects intracellular trafficking between cellular membrane compartments [27]. All these processes are important for immune cell functions.

In fact, recent studies have shown that some widely prescribed mevalonate‐pathway‐inhibiting drugs not only alleviate the severity of targeted diseases but also exert effects on the immune system [28, 29]. Statins, for example, are used to lower cholesterol levels and reduce the risk of cardiovascular disease by inhibiting the rate‐limiting enzyme HMG‐CoA reductase in the mevalonate pathway [30, 31]. As they can modulate immune cell function in various ways depending on the cell type and condition, they generally show immunosuppressive effects [32, 33, 34]. Aminobisphosphonates are the most used drugs to treat a wide array of disorders of bone fragility, including osteoporosis [35, 36]. Intravenous administration of zoledronate induces transient fever in most patients [37]. The inflammatory reaction might be caused by the activated Vδ2 T cells.

The importance of mevalonate metabolism on the immune response also manifests in an autoinflammatory disease. A deficiency of mevalonate kinase, which phosphorylates mevalonate in the isoprenoid biosynthesis pathway, causes hyper‐IgD syndrome (HIDS). The syndrome is characterised by recurring attacks of fever and other inflammatory symptoms such as joint pain, swollen lymph nodes, skin rash, headaches and abdominal pain [38]. An accumulation of non‐processed mevalonate in HIDS has been shown to cause an inflammatory phenotype in innate immune cells [39].

In this study, we aim to assess the effect of mevalonate pathway inhibition in Vδ2 T cells on their immune function. We showed that the intrinsic mevalonate metabolism fuels cytokine and cytotoxic granule production by these cells. Especially protein prenylation and signal transduction, downstream mevalonate pathway, are important for the proinflammatory and cytotoxic functions of Vδ2 T cells. This study provides insight into the off‐target effects caused by drugs such as aminobisphosphonates and statins on the proper functioning of Vδ2 T cells, as well as into the challenges of the common zoledronate‐based Vδ2 T cell expansion protocol.

## 2 Results

### 2.1 In Vitro Inhibition of the Mevalonate Pathway Compromises TNF and IFN‐γ Production by Vδ2 T Cells

**FIGURE 1:**
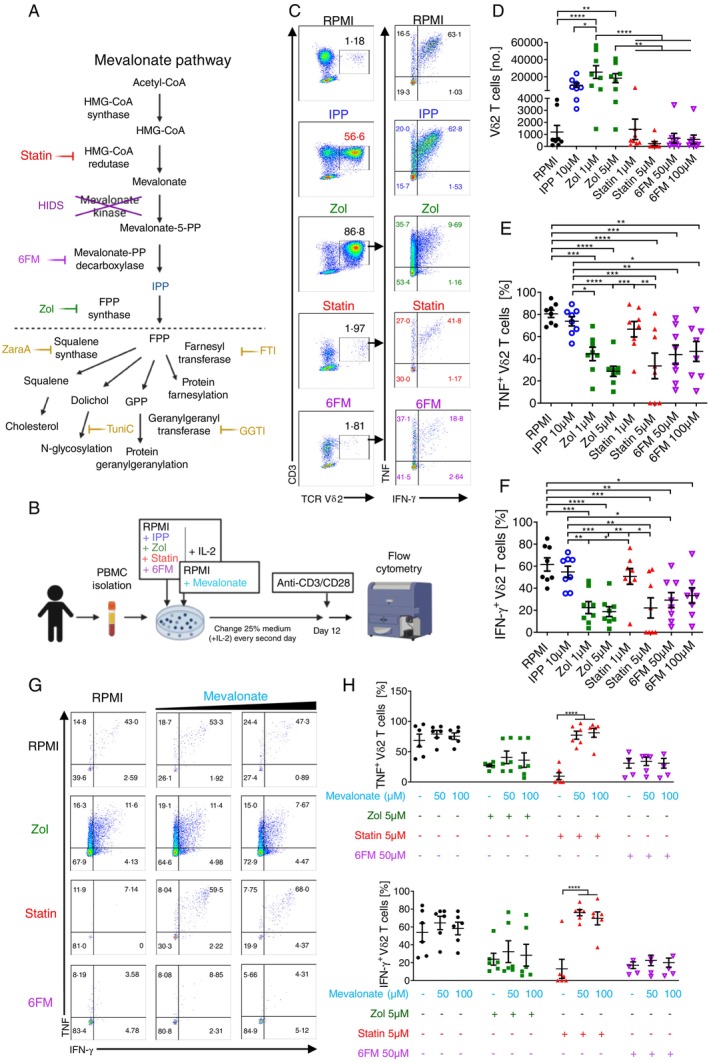
In vitro inhibition of mevalonate pathway results in compromised TNF and IFN‐γ production by Vδ2 T cells. (A) Schematic representation of the mevalonate pathway, the inhibitors used in the study and downstream biological processes: Fluvastatin (Statin) inhibits HMG‐CoA reductase; Hyper IgD syndrome (HIDS) is caused by deficiency in mevalonate kinases; 6‐fluoromevalonate (6FM) inhibits mevalonate‐5‐PP decarboxylase; zoledronate (Zol) inhibits FPP synthase; zaragozic acid (ZaraA) inhibits squalene synthase; Tunicamycin (TuniC) alters N‐linked glycosylation of proteins; GGTI 2133 inhibits geranylgeranyl transferase; and FTI 277 (FTI) inhibits farnesyl transferase. (B) Experimental setup for in vitro inhibition of mevalonate pathway and rescue experiment with mevalonic acid. (C–F) Flow cytometry analysis of Vδ2 T cells in 12‐days PBMC cultures treated with indicated inhibitors (Mean ± SEM, *n* = 8): (C) representative dot plots showing percentage of Vδ2 T cells in PBMC cultures (left column) and percentage of TNF^+^ and IFN‐γ^+^ Vδ2 T cells (right column); (D) cumulative numbers of Vδ2 T cells; (E) cumulative percentages of TNF^+^ and (F) IFN‐γ^+^ Vδ2 T cells. (G) Representative FACS plots showing percentage of cytokine‐producing Vδ2 T cells in PBMC cultures treated with indicated inhibitors in the presence or absence of mevalonic acid (50 or 100 μM). (H) Cumulative percentage of TNF^+^ and IFN‐γ^+^ Vδ2 T cells in PBMC cultures treated with indicated inhibitors in the presence or absence of mevalonic acid (Mean ± SEM, *n* = 6). Each dot represents one donor in (D–F) and (H), repeated measures one‐way ANOVA followed by Tukey's multiple comparisons test, *p value < 0.05. 6FM: 6‐fluoromevalonate. Created with Biorender. FPP: farnesyl pyrophosphate; GPP: geranyl pyrophosphate; HIDS: hyper IgD syndrome; HMG‐CoA: β‐hydroxy β‐methylglutaryl‐coenyzme A; mevalonate‐5‐PP decarboxylase: mevalonate diphosphate decarboxylase.

To determine the effect of mevalonate pathway inhibition in Vδ2 T cells on their pro‐inflammatory cytokine production, we incubated PBMCs from healthy donors with zoledronate (Zol) or other pharmacological inhibitors targeting different enzymes in the mevalonate pathway: fluvastatin (Statin) and the mevalonate‐PP decarboxylase inhibitor 6‐fluoromevalonate (6FM), as well as IPP and non‐treated cultures (RPMI alone) as controls, in the presence of IL‐2 (Figure 1A,B). After 12 days of culture, consistent with numerous in vitro Vδ2 T cell expansion protocols for cancer therapy [14, 40, 41, 42, 43, 44, 45], we assessed the proliferation and cytokine production capacity of Vδ2 T cells by flow cytometry upon anti‐CD3 and anti‐CD28 restimulation (Figure 1C–F and Figure S1A). Anti‐CD3/CD28 antibodies were chosen for the cytokine intracellular staining instead of phosphoantigen or PMA and ionomycin in order to address the cytokine production potential of Vδ2 T cells independently of butyrophilin expression in the culture, which is required for the activation of Vδ2 T cells by phosphoantigens [11, 46, 47], but under more physiological conditions than PMA/ionomycin. Furthermore, anti‐CD3/CD28 stimulation allowed us to simultaneously analyse conventional CD4 and CD8 T cells in the culture. Consistent with previous studies [13, 14], the numbers of Vδ2 T cells in IPP‐ and zoledronate‐treated cultures increased significantly (Figure 1C,D). The number of Vδ2 T cells in 6‐fluoromevalonate‐treated cultures was comparable to that in non‐treated cultures, while the number of Vδ2 T cells in fluvastatin‐treated cultures was reduced at the higher dose (5 μM) (Figure 1C,D). This could be due to impaired proliferative capacity of Vδ2 T cells or increased cytotoxicity upon treatment with higher doses of statin (Figures 1D and S1B,C). Cytokine production assessment revealed that Vδ2 T cells incubated in RPMI medium alone or treated with IPP were potent producers of TNF and IFN‐γ, while the percentages of TNF‐ and IFN‐γ‐producing Vδ2 T cells significantly decreased in the presence of zoledronate (Figure 1C,E,F). The cytokine production capacity by Vδ2 T cells was consistently reduced in longer term cultures, namely 14, 18 and 21 days (Figure S1D).

Proliferation capacity and effector function vary between naïve and memory T cell subsets [48]. Although zoledronate treatment (in comparison to IL‐2 alone) polarised Vδ2 T cells more toward an effector memory phenotype (CD45RA‐CD27‐), shown to readily produce cytokines and exert effector functions [49], we still observed an overall decrease in cytokine production by Vδ2 T cells, indicating that their decreased function may be independent of the alteration of memory phenotype (Figure S1E).

The stimulatory effect of zoledronate on Vδ2 T cells in PBMC cultures has been mainly attributed to monocytes [10, 50, 51] and dendritic cells [52] with the inhibitory effect of neutrophils [53] and no involvement of conventional T cells [10]. To identify whether the observed decrease in cytokine production upon zoledronate treatment is due to the direct effect of zoledronate on Vδ2 T cells instead of an indirect effect induced by the drug through other cellular components in the PBMC culture, we purified Vδ2 T cells from PBMCs of healthy donors and incubated the cells with or without different concentrations of zoledronate in the presence of IL‐2 for up to 6 days. Although the purification efficiency varied in Vδ2 T cell cultures (Figure S1F), consistently with our original findings, we observed the reduction of TNF and IFN‐γ production by Vδ2 T cells starting from day 2 of incubation with zoledronate (Figure S1G) even in over 99% pure cultures (Figure S1H), indicating zoledronate can directly affect Vδ2 T cells. At day 6 of culture, the cytokine production capacity further decreased together with perforin production, but the cell viability also started to decrease, which potentially also contributed to the Vδ2 T cell dysfunction.

Similarly, other mevalonate pathway inhibitors such as 6‐fluoromevalonate and higher doses of fluvastatin (Figure 1C,E,F) and atorvastatin (Figure S1I) also reduced the percentages of live TNF‐ and IFN‐γ‐producing Vδ2 T cells. To disentangle whether the reduction of cytokine production by Vδ2 T cells is the result of long‐term mevalonate pathway inhibition or the effect of inhibition on cytokine production only during restimulation, we expanded Vδ2 T cells in PBMC cultures with IPP and IL‐2 for 12 days and incubated them with mevalonate pathway inhibitors only during the cytokine assay (4 h restimulation with anti‐CD3/CD28). The cytokine production was not significantly affected by mevalonate pathway inhibition during the cytokine assay (Figure S1J), suggesting that the impaired functional response of Vδ2 T cells is the result of longer incubation with the inhibitors during the culturing period of Vδ2 T cells. We also examined αβ T cells in our cultures and found that zoledronate and fluvastatin treatment reduced the numbers of IFN‐γ‐producing CD4 T cells, but only fluvastatin treatment affected cytokine production by CD8 T cells (Figure S1K,L). These data indicate that in vitro inhibition of mevalonate metabolism leads to impaired cytokine production by Vδ2 and conventional T cells.

To validate that the effect of the drugs on cytokine production is due to the inhibition of mevalonate metabolism, we supplemented PBMC cultures with mevalonic acid (Figure 1G,H). Mevalonic acid alone had no effect on Vδ2 T cells, while the supplementation of mevalonic acid to fluvastatin‐treated cultures restored the viability and TNF and IFN‐γ production by live‐gated Vδ2 T cells (Figures 1G,H and S1C) as well as conventional CD4 and CD8 T cells (data not shown). This indicates that the decreased cytokine production by live cells and increased percentage of overall cell death in fluvastatin‐treated cultures are the effects of the mevalonate deficiency upon the blockage of the pathway and not directly caused by the drug cytotoxicity. As expected, mevalonate addition to the PBMC cultures treated with downstream inhibitors: zoledronate or 6‐fluoromevalonate had no effect on Vδ2 T cells (Figure 1G,H). Altogether, these data indicate that the effect of the inhibitors on Vδ2 T cell function is mediated by the suppression of mevalonate metabolism.

### 2.2 In Vivo Inhibition of the Mevalonate Pathway Compromises TNF and IFN‐γ Production by Vδ2 T Cells

**FIGURE 2:**
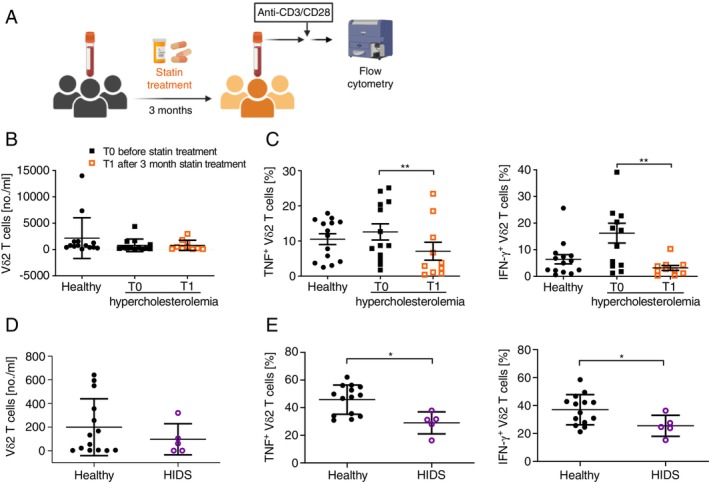
In vivo inhibition of mevalonate pathway results in compromised TNF and IFN‐γ production by Vδ2 T cells. (A) Schematic representation of flow cytometry analysis of peripheral blood from patients with hypercholesterolemia before and after 3‐months of statin treatment (atorvastatin or rosuvastatin). (B) Number of Vδ2 T cells and (C) percentage of TNF^+^ and IFN‐γ^+^ Vδ2 T cells in healthy individuals and patients with hypercholesterolemia before and after 3‐months of statin treatment (Mean ± SEM, *n* = 13: Patients T0; *n* = 10 patients T1; *n* = 14: Healthy donors; Mann–Whitney test: Patients and healthy donors; Wilcoxon test: Before and after treatment, *p value < 0.05). (D) Number of Vδ2 T cells and (E) percentage of TNF^+^ and IFN‐γ^+^ Vδ2 T cells in healthy individuals and patients with Hyper IgD syndrome, (Mean ± SEM, *n* = 5: Patients; *n* = 14: Healthy donors; Mann–Whitney test, *p value < 0.05) Created with Biorender.

To verify whether our in vitro observations translate to the in vivo settings, we performed a longitudinal cohort study on patients with hypercholesterolemia who were prescribed statin therapy (atorvastatin or rosuvastatin) (Table S1). We assessed the numbers and cytokine production capacity of Vδ2 T cells from these patients before and after 3 months of statin treatment (Figure 2A). The numbers of Vδ2 T cells in patients were comparable to those in healthy donors (Figure 2B). In consistency with our in vitro findings, the percentage of TNF‐ and IFN‐γ‐producing Vδ2 T cells markedly decreased after 3 months of statin treatment (Figure 2C). Statin treatment also affected the cytokine production capacity of CD4 and CD8 T cells (Figure S2A,B) but to a lesser extent than that of Vδ2 T cells (Figure 2C).

Furthermore, we recruited patients with hyper‐IgD syndrome who have a deficiency in mevalonate kinase (Figure 2D,E). Consistently, we observed no significant change in Vδ2 T cell numbers but a reduced proportion of TNF‐ and IFN‐γ‐producing Vδ2 T cells in patients compared to healthy controls. Yet, this condition had no effect on αβ T cells (Figure S2C,D). Altogether, our in vitro and in vivo data show that mevalonate metabolism plays an important role in cytokine production by Vδ2 T cells.

### 2.3 Zoledronate Upregulates Exhaustion Marker TIM3 on Vδ2 T Cells

**FIGURE 3:**
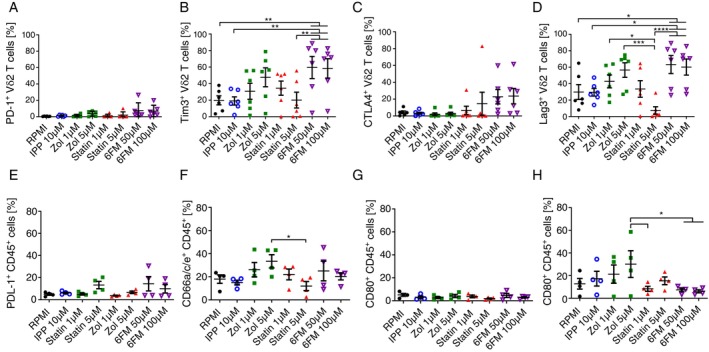
Zoledronate upregulates exhaustion marker TIM3 on Vδ2 T cells. (AH) Flow cytometry analysis of Vδ2 T cells in 12‐days PBMC cultures treated with indicated inhibitors: Cumulative percentage of (A) PD‐1^+^, (B) TIM3^+^, (C) LAG3^+^ and (D) CTLA4^+^ Vδ2 T cells (Mean ± SEM, *n* = 6); Cumulative percentage of (E) PDL1^+^, (F) CD66a/c/e^+^, (G) CD80^+^ and (H) CD86^+^ expression on CD45^+^ cells (Mean ± SEM, *n* = 4). Each dot represents one donor (repeated measures one‐way ANOVA followed by Tukey's multiple comparisons test, *p value < 0.05).

An underlying mechanism leading to compromised cytokine production may be, in large part, the exhaustion of Vδ2 T cells in long‐term cultures. To determine whether inhibition of mevalonate metabolism causes exhaustion, we assessed the transcript and protein expression levels of immune‐suppressive checkpoint receptors such as PD‐1, TIM3, CTLA‐4, and LAG3 [54, 55, 56, 57] on the Vδ2 T cells in our cultures (Figures 3A–D and S3). Our RNA‐sequencing (RNA‐seq) data revealed a significant upregulation of *HAVCR2*, the TIM3 transcript, in Vδ2 T cells from zoledronate‐treated cultures (Figure S3A). Furthermore, zoledronate treatment increased the numbers of TIM3‐ and LAG3‐expressing Vδ2 T cells compared to the IPP and RPMI alone conditions (Figure 3A–D). However, unlike in the 6‐fluoromevalonate condition, this upregulation was not significant. Although the enhanced expression of checkpoint receptors is a strong indicator of immune cell exhaustion, their inhibitory effect is only exerted in the presence of their ligands. Therefore, we also assessed the expression of the checkpoint receptor ligands: PDL1, CD66a/c/e^+^, CD80, and CD86 on PBMCs in our cultures [58, 59, 60] (Figure 3E–H). A higher proportion, yet statistically not significant, of zoledronate‐treated PBMCs expressed TIM3 and CTLA‐4 ligands: CD66a/c/e and CD86, respectively (Figure 3F,H). Furthermore, we also analysed the transcript levels of other key exhaustion‐associated molecules such as *TBX21*, *EOMES*, *TOX*, and *CD39* [61, 62] in our RNA‐seq data. Mevalonate pathway inhibition did not significantly affect the transcript levels of these genes in Vδ2 T cells (Figure S3B). However, the *CD39* expression in Vδ2 T cells was significantly increased upon IPP treatment but not significantly increased by Zol treatment compared to the RPMI condition, suggesting that this could be a result of cell activation in both conditions. Therefore, exhaustion mechanisms may partially explain the reduced cytokine production capacity of zoledronate‐expanded Vδ2 T cells. However, we did not observe a coherent pattern of the expression of exhaustion markers in all mevalonate pathway inhibitor‐treated conditions.

### 2.4 Mevalonate Pathway Inhibition Induces Transcriptome Changes in Vδ2 T Cells

**FIGURE 4:**
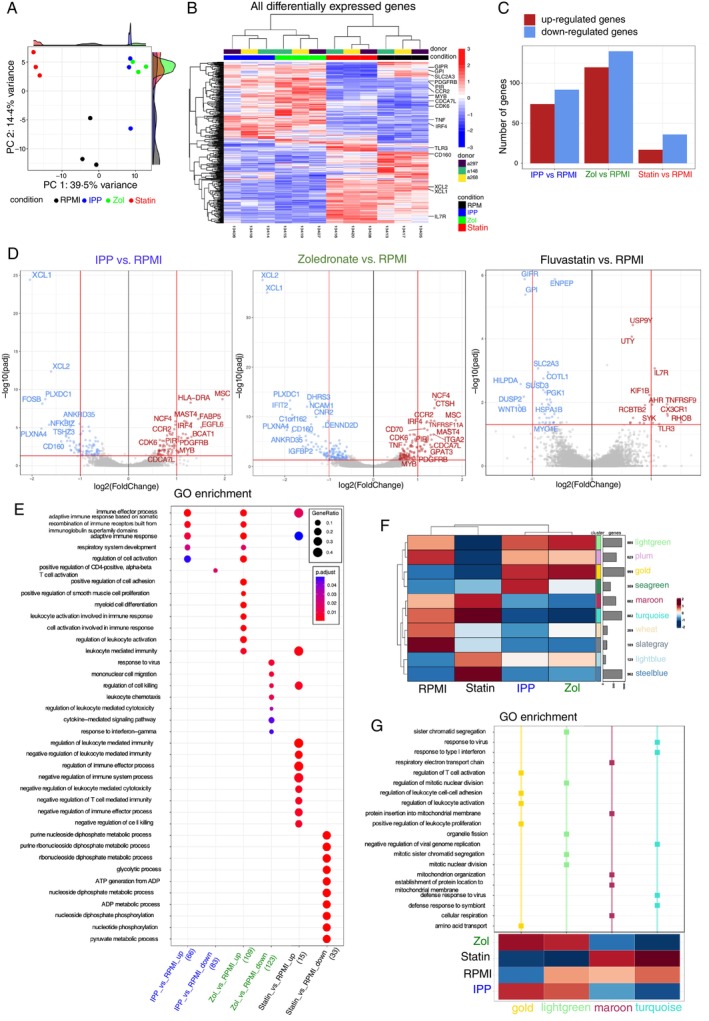
Mevalonate pathway inhibition induces transcriptome changes in Vδ2 T cells. (A–G) RNA‐seq analysis of Vδ2 T cells isolated from 12‐days‐PBMC cultures treated with IPP, zoledronate (Zol), fluvastatin (Statin) or RPMI alone (*n* = 3). (A) Principal component analysis coloured by experimental conditions: RPMI (black), IPP (blue), Zol (green) and Statin (red). (B) Clustered heatmap showing 479 differentially regulated genes between RPMI, IPP, Zol and Statin conditions. Selected genes are annotated. (C) Number of differentially expressed genes in IPP, Zol and Statin conditions versus RPMI. (D) Volcano plot demonstrating differentially upregulated and downregulated transcripts. Grey points indicate genes with *p* values > 0.05 and log_2_fold change > sigFC = −2 and < sigFC = 2. Blue points indicate genes with *p* values < 0.05 and log_2_fold change < sigFC = −2. Red points indicate genes with *p* values < 0.05 and log_2_fold change > sigFC = 2. (E) Enrichment dot plots for IPP over RPMI, Zol over RPMI and Statin over RPMI treatments, showing most significantly enriched GO terms. The topmost enriched terms with adjusted *p* values ≤ 0.1 are demonstrated. (F) hCoCena Integrated group fold change (GFC) heat map showing hierarchical clustering and gene modules identified by hCoCena analysis for the RPMI, IPP, zol and statin‐treated groups. Numbers and bar‐plots on the right side reflect the sizes of the modules. (G) Functional enrichment of hCoCena‐derived modules using the GO gene set database. Selected top terms were visualised.

We further determine the effect of mevalonate pathway inhibition on whole transcriptome and chromatin landscapes by performing RNA‐seq along with the Assay for Transposase‐Accessible Chromatin using Sequencing (ATAC‐seq) on Vδ2 T cells sorted from zoledronate‐, IPP‐ and non‐treated PBMC cultures without restimulation (Figures 4 and S4). Additionally, we sorted Vδ2 T cells from fluvastatin‐treated cultures for comparison as another mevalonate pathway inhibitor. Principal component analysis of RNA‐seq data revealed clustering of samples based on culturing conditions, where IPP‐ and zoledronate‐expanded Vδ2 T cells showed the most similarity in the transcriptional programs (Figure 4A,B). The highest number of transcripts was differentially up‐ and down‐regulated in IPP‐ and zoledronate‐treated Vδ2 T cells when compared to the Vδ2 T cells from RPMI‐treated cultures (Figures 4C,D and S4A). Among mutually upregulated transcripts in IPP‐ and zoledronate‐treated Vδ2 T cells vs. RPMI alone, we found genes related to the regulation of cell cycle (e.g., *CDK6*, *CDCA7L*), proliferation (e.g., *PDGFRB*, *MYB*) and replication (e.g., *PIR*) (Figure 4B,D). Furthermore, some transcripts related to immune functions were both mutually up‐regulated (e.g., CCR2, IRF4), while others were mutually down‐regulated (e.g., XCL, CD160) in IPP‐ and zoledronate‐treated Vδ2 T cells (Figure 4B,D).

While we did not detect significant differences in *IFNG* expression between all conditions based on differential expression analysis, the *TNF* transcript was upregulated in zoledronate‐treated cells (Figure 4B,D). Gene ontology (GO) enrichment analysis further revealed the up‐regulation of immune effector processes and adaptive immune response in IPP‐ and zoledronate‐treated compared to RPMI‐treated Vδ2 T cells (Figure 4E). Genes that were upregulated in zoledronate‐treated Vδ2 T cells also showed enrichment in positive regulation of cell activation involved in immune response, leukocyte mediated immunity and cell–cell adhesion (Figure 4E), while genes downregulated in zoledronate‐treated but not in IPP‐treated Vδ2 T cells showed enrichment in processes including cell migration, leukocyte cytotoxicity and cytokine signalling (Figure 4E). Among all conditions, fluvastatin‐treated Vδ2 T cells presented the lowest number of up‐regulated transcripts versus RPMI, which were mainly related to immune function (e.g., *IL‐7R*, *TLR3*) and were enriched in GO terms such as negative regulation of immune effector processes, including leukocyte mediated immunity and cytotoxicity (Figures 4B–D and S4A). On the other hand, significantly down‐regulated transcripts in fluvastatin‐treated Vδ2 T cells were in large proportion related to metabolic processes (e.g., *GIPR*, *GPI*, *SLC2A3*) and showed enrichment in GO terms related to many metabolic processes such as glycolysis and phosphorylation (Figure 4B,D). The commonly upregulated genes in zoledronate‐ and fluvastatin‐treated Vδ2 T cells showed enrichment in GO pathways related to leukocyte mediated immunity, regulation of immune effector processes and adaptive immune response (Figure 4E). To further characterise differentially mobilised functional gene modules in Vδ2 T cells between conditions, horizontal construction of co‐expression networks and analysis (hCoCena) was applied (Figures 4F,G and S4B) [63]. hCoCena allows the analysis of all conditions at the same time, identifying common and condition‐specific co‐expressed genes. The resulting network consisted of 5786 nodes (genes), connected by 63 226 edges (co‐expression relationships) (Figure S4B). Ten modules were identified using unsupervised Leiden clustering, highlighted by specific colours, representing groups of genes with similar expression patterns (Figure 4F, left), while the heatmap visualises the modules' mean group fold changes (GFCs) across all conditions (Figure 4F, right). The genes grouped in each module were used for GO enrichment analysis. Consistent with the phenotype observed, this analysis revealed that gene modules up‐regulated in IPP and zoledronate‐treated Vδ2 T cells (gold) were enriched in GO terms related to leukocyte activation and adhesion and amino acid transport, as well as mitotic nuclear division and organelle fission (lightgreen) (Figure 4G). In contrast, the down‐regulated modules in these conditions showed enrichment in GO terms associated with mitochondrial function (maroon) and response to viruses (turquoise) (Figure 4G). The down‐regulated modules in fluvastatin‐treated Vδ2 T cells showed enrichment in processes related to cell division (lightgreen) (Figure 4G). Furthermore, the close analysis of the transcription factor transcripts that are known to directly regulate *IFNG* and *TNF* expression, such as *TBX21*, *STAT*, *NFAT*, and so forth, could not explain the effect of inhibitors on the cytokine levels in Vδ2 T cells (Figure S4B). Altogether, the RNA‐seq data unravelled the modulatory effect of mevalonate metabolism on the transcriptome of Vδ2 T cells. We further assessed whether mevalonate pathway inhibition affects the transcriptome by modulating chromatin accessibility. The ATAC‐seq analysis demonstrated that the chromatin accessibility of differentially expressed transcripts, including *TNF* and *IFNG* loci, was not significantly affected in IPP‐, zoledronate‐ and fluvastatin‐ vs. RPMI alone‐treated Vδ2 T cells (Figure S4C). Altogether, the next‐generation sequencing analysis revealed global changes in the Vδ2 T cell transcriptome upon mevalonate pathway inhibition. However, there were few commonalities at the transcript level between zoledronate‐ and fluvastatin‐treated cells, indicating that these inhibitors have distinct effects on the Vδ2 T cell transcriptome, which cannot fully explain the commonly seen impairment of cytokine production.

### 2.5 Zoledronate and Statin Distinctively Affect the Metabolic Profile of Vδ2 T Cells

Our RNAseq analysis revealed the down‐regulation of a large proportion of transcripts related to metabolic processes, including glycolytic processes (e.g., *GIPR*, *GPI*, *SLC2A3*) in Vδ2 T cells from fluvastatin‐treated PBMC cultures (Figure 4B,D). To address glucose metabolism in Vδ2 T cells upon mevalonate pathway inhibition, we employed SCENITH metabolic profiling [64] on 12 day PBMC cultures treated with mevalonate pathway inhibitors. Glycolytic capacity of Vδ2 T cells was increased upon 6‐fluoromevalonate treatment, while the fatty acid and amino acid oxidation (FAO&AAO) capacity was reduced in higher concentrations of statin when compared to RPMI condition (Figure S4D). Therefore, the changes in energy metabolism of Vδ2 T cells upon mevalonate pathway inhibition may not be the direct cause of the decreased functionality of the cells.

### 2.6 Inhibition of Protein Prenylation Impairs TNF and IFN‐γ Production by Vδ2 T Cells

**FIGURE 5:**
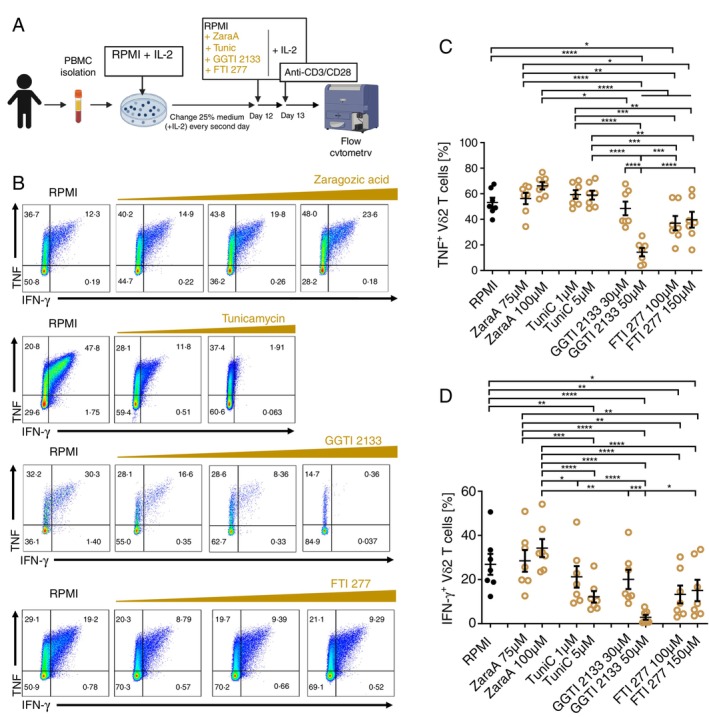
Inhibition of protein prenylation impairs TNF and IFN‐γ production by Vδ2 T cells. (A) Experimental setup for the in vitro inhibition of downstream mevalonate pathways. PBMCs were first expanded with IPP in the presence of IL‐2 for 12 days. On day 12, the indicated inhibitors: Zaragozic acid (75 and 100 μM), tunicamycin (1 and 5 μM), geranylgeranyl transferase inhibitor (GGTI2133; 30 and 50 μM) and farnesyl transferase inhibitor (FTI277; 100 and 150 μM) were added for overnight incubation. (B–D) Flow cytometry analysis of Vδ2 T cells in PBMC cultures treated as described above (Mean ± SEM, *n* = 7). (B) Representative dot plots showing percentage of TNF^+^ and IFN‐γ^+^ Vδ2 T cells in PBMC cultures; (C) cumulative percentage of TNF^+^ and (D) IFN‐γ^+^ Vδ2 T cells in PBMC cultures. Each dot represents one donor (repeated measures one‐way ANOVA followed by Tukey's multiple comparisons test, *p value < 0.05). Created with Biorender.

The transcriptome changes that we observed in Vδ2 T cells in the presence of inhibitors are most likely the secondary effect of the impaired biological processes downstream of the mevalonate pathway (Figure 1A). To further decipher which of these processes affect the cytokine production by Vδ2 T cells, we pre‐treated PBMC cultures with IPP and IL‐2, as this condition expands the Vδ2 T cells but does not impair mevalonate metabolism and does not affect the cytokine production capacity of Vδ2 T cells (Figures 1 and 5). After 12 days of expansion, the cultures were incubated for an additional 24 h in the presence of different downstream mevalonate pathway inhibitors: zaragozic acid (ZaraA), tunicamycin (TuniC), geranylgeranyltransferase inhibitors (GGTI) and farnesyltransferase inhibitors (FTI) and the cytokine production by Vδ2 T cells was assessed (Figure 5). Zaragozic acid, which blocks cholesterol synthesis by inhibiting squalene synthesis (Figure 1A), did not significantly affect the number of cytokine‐producing Vδ2 T cells (Figure 5B–D). This is the opposite phenotype to the one caused by zoledronate, statins, and 6‐fluoromevalonate, which inhibit cytokine production (Figure 1C,E,F). To evaluate whether cholesterol levels in Vδ2 T cells are indeed affected by the inhibitors, we performed filipin staining (Figure S5A,B). Interestingly, the surface and intracellular cholesterol levels were not affected in Vδ2 T cells; therefore, it is unlikely that fluvastatin, zoledronate, and 6‐fluoromevalonate affect TCR signalling through cholesterol depletion.

In the presence of tunicamycin, which blocks the N‐glycosylation process (Figure 1A), the percentage of IFN‐γ‐producing Vδ2 T cells significantly decreased while the TNF‐producing cells slightly increased (Figure 5B–D). This is consistent with the fact that IFN‐γ, but not TNF, undergoes N‐glycosylation [65, 66]. Since only IFN‐γ production by Vδ2 T cells is affected by disruption of N‐glycosylation, there are additional regulatory mechanisms that contribute to the Vδ2 T cell phenotype caused by fluvastatin, zoledronate and 6‐fluoromevalonate.

Finally, we tested protein prenylation, which is another posttranslational modification found on many signalling proteins (Figure 1A). Inhibition of protein prenylation by farnesyltransferase inhibitor FTI277 and geranylgeranyltransferase inhibitors: GGTI2133, GGTI298 and GGTI286 (Figure 1A) decreased both TNF and IFN‐γ production by Vδ2 T cells (Figure 5B–D and Figure S5C). These data indicate that protein prenylation, especially protein geranylgeranylation, and the downstream mevalonate pathway are important for TNF and IFN‐γ production by Vδ2 T cells.

### 2.7 In Vitro Inhibition of the Mevalonate Pathway Leads to Dysregulation of Signal Transduction Pathways in Vδ2 T Cells

**FIGURE 6:**
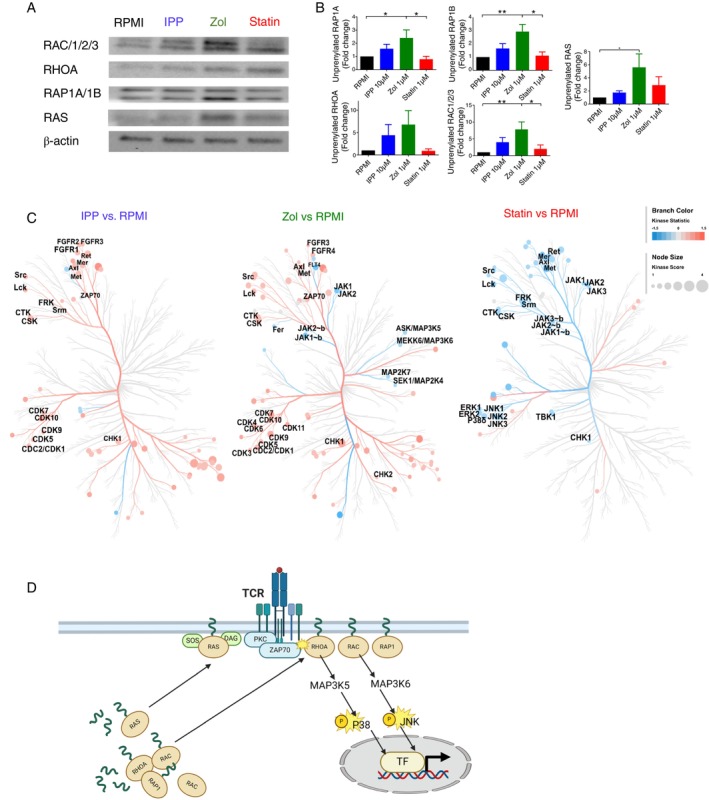
In vitro inhibition of the mevalonate pathway leads to dysregulation of signal transduction pathways in Vδ2 T cells. (A) Representative Western Blot image showing enrichment of unprenylated small G proteins: RAC, RHOA, RAP1 and RAS in the cytosol of Vδ2 T cells isolated from 12‐days‐PBMC cultures treated with the indicated inhibitors. (B) Quantification of cytosolic small G proteins in Western Blot using imageJ. Fold change was calculated over RPMI condition (Mean ± SEM, *n* = 5) (repeated measures one‐way ANOVA followed by Tukey's multiple comparisons test, *p value < 0.05). (C) PamGene analysis of kinase activity in Vδ2 T cells isolated from PBMCs cultured 12 days with IPP, zoledronate, fluvastatin or RPMI alone and restimulated for 10 min with anti‐CD3/CD28 (*n* = 7): Kinome tree showing kinases with up‐ and down‐regulated activity in Vδ2 T cells. (D) Schematic representation of the molecular mechanisms by which mevalonate metabolism fuels Vδ2 T cell function. Briefly, FPP generated in the mevalonate pathway serves for protein prenylation. Among prenylated proteins are small GTPases which when prenylated anchor to the cell membrane where they interact with various receptors including T cell receptor (TCR). Upon TCR stimulation small GTPases are activated and transduce the signal to the nucleus by activating downstream signalling pathways such as MAPK signalling. This results in transcription factors recruitment to the nucleus and induction of effector gene expression. Mevalonate pathway inhibition results in impaired protein prenylation and consequently compromised signal transduction upon TCR activation resulting in impaired Vδ2 T cell function. Created with Biorender. FPP: farnesyl pyrophosphate; GGPP: Geranylgeranyl pyrophosphate; JNK: c‐Jun N‐terminal kinases; MAP3K: Mitogen‐Activated Protein Kinase Kinase Kinase; Rac: Ras‐related C3 botulinum toxin substrate; RAP1, Ras‐related protein 1; Ras: Rat sarcoma; RHOA: Ras homologue family member A; TF: transcription factor.

Protein prenylation has been shown to play a role in signalling events important for T cell activation [67]. For example, small GTPases are signal transducing molecules activated in response to TCR stimulation [68, 69]. When prenylated, they anchor to the cellular membranes, where they can interact with various receptors [70]. To verify whether protein prenylation of small GTPases such asRAC, RHOA, RAP1 and RAS is indeed compromised in Vδ2 T cells upon mevalonate inhibition, we extracted the cytosolic proteins from purified Vδ2 T cells derived from zoledronate‐, fluvastatin‐, IPP‐ and RPMI alone‐treated PBMC cultures and performed Western Blot analysis (Figure 6A,B). A significant accumulation of unprenylated small G proteins such as RAC, RAP1A/B and RAS was observed in the cytosol fraction of zoledronate‐expanded Vδ2 T cells compared to untreated (RPMI alone condition). Fluvastatin treatment resulted in a slight increase of unprenylated RAS while IPP‐expanded Vδ2 T cells accumulated unprenylated RAC and RHOA, yet these changes were not significant (Figure 6A,B). This data indicate that protein prenylation in Vδ2 T cells is affected by mevalonate pathway modulation, where zoledronate inhibition showed the strongest effect.

Many signalling events downstream of small GTPases, such as MAP kinases: ERK, P38 and JNK signalling, are important for many effector functions of T cells, including cytokine production [71]. To investigate whether these signalling pathways are indeed affected due to the compromised protein prenylation upon mevalonate pathway inhibition, we performed the PamGene global kinase activity assay on TCR‐restimulated Vδ2 T cells from our PBMC cultures (Figure 6C,D and Figure S6). The PamGene technology allows the determination of the activity of numerous kinases based on the phosphorylation status of the target phosphosites. To that end, Vδ2 T cells were purified from the 12‐day cultures and stimulated with anti‐CD3/CD28 for 10 min prior to the kinome assessment. We found that among all treatment conditions, zoledronate‐ and fluvastatin‐treatment resulted in a higher number of phosphosites with decreased phosphorylation than IPP‐treatment when compared to RPMI (Figure S6A), while IPP affected the smallest number of phosphosites compared to the RPMI condition (Figure S6A). By performing the global kinase activity analysis, we observed the highest number of kinases with upregulated activity in the IPP and zoledronate conditions and the highest number of kinases with downregulated activity in the statin condition (Figures 6C,B and S6B). In particular, both IPP‐ and zoledronate increased the activity of kinases related to proliferation, differentiation and cell cycle regulation, such as AXL, a receptor kinase that binds to GAS6 and regulates cell proliferation and survival [72]; FGFRs, receptor kinases important for mitogenesis and differentiation [73]; and CHKs, kinases that act as key regulators of the cell cycle [74] (Figure 6C). This is in line with the observed high proliferation rate of Vδ2 T cells in these cultures (Figure 1C,D). In addition, proximal TCR signalling was enhanced in IPP‐ and Zol‐treated Vδ2 T cells, as illustrated by the upregulation of LCK, ZAP70, SCR activity compared to RPMI alone, and no significant difference was found in the activity of these kinases between IPP and zoledronate treatment (Figure 6C). This suggests that TIM3 signalling, which interferes with the proximal TCR signalling [75, 76], might not be the main driving mechanism of the impaired Vδ2 T cell function as suggested by the RNA‐seq and flow cytometry results (Figures 3 and S3). However, the activities of serine/threonine kinases downstream of small GTPases, such as ASK/MAP3K5 and SEK1/MAP2K4, were downregulated upon zoledronate treatment, which was not observed in the IPP condition (Figure 6C). Similarly, in fluvastatin‐treated cells, the activities of kinases such as ERK, P38 and JNK were reduced in response to short TCR activation, indicating that effector signalling cascades downstream of GTPases are impaired upon mevalonate pathway inhibition. Furthermore, most of the tyrosine receptor kinases, which are important for the initiation of receptor signalling such as LCK, LTK and MET, were affected in the fluvastatin‐treated condition (Figure 6C), indicating that the kinases upstream of GTPases are also highly affected. Contrary to IPP‐ and zoledronate‐treated Vδ2 T cells, the kinases regulating proliferation and differentiation, such as TBK1 and CHK1, were downregulated in fluvastatin‐treated cultures, in line with the decreased proliferation of Vδ2 T cells in this condition (Figure 1C,D). Interestingly, our analysis did not reveal kinases commonly regulated by the two pharmacological inhibitors, consistent with their distinct effect on the prenylation status of small G proteins (Figure 6A,B). Overall, manipulation of the mevalonate pathway results in dysregulation of the kinome of Vδ2 T cells, yet the effect of different inhibitors is specific (Figure 6D) in which zoledronate treatment abrogated signalling directly downstream of GTPases while fluvastatin treatment impaired the signalling of both upstream and downstream of GTPases in Vδ2 T cells.

### 2.8 Mevalonate Metabolism Is Important for Cytotoxic Properties of Vδ2 T Cells

**FIGURE 7:**
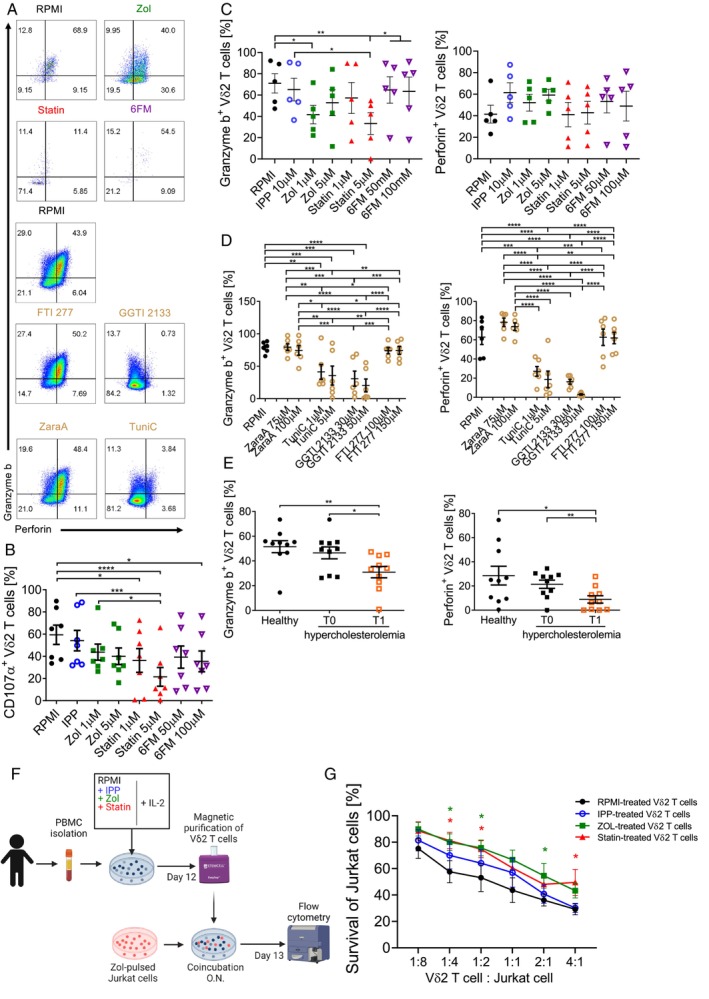
Mevalonate metabolism is important for cytotoxic properties of Vδ2 T cells. (A–D) Flow cytometry analysis of Vδ2 T cells in PBMC cultures treated as previously with indicated inhibitors (Mean ± SEM, (A, C) *n* = 5; (B): *n* = 7, (D) *n* = 6): (A) representative dot plots showing percentage of granzyme B^+^ and perforin^+^ Vδ2 T cells; cumulative percentage of (B) CD107a^+^, (C, D), granzyme B^+^ (left panel) and perforin^+^ (right panel) Vδ2 T cells in PBMC cultures after incubation with indicated inhibitors (repeated measures one‐way ANOVA followed by Tukey's multiple comparisons test, *p value < 0.05). (E) Cumulative percentage of granzyme B^+^ and perforin^+^ Vδ2 T cells in patients with hypercholesterolemia before and after 3‐months of statin treatment (Mean ± SEM, *n* = 10: Patients T0, T1; *n* = 10: healthy donors); (Mann–Whitney test: patients and healthy donors; Wilcoxon test: before and after treatment, *p value < 0.05). (B–E) Each dot represents one donor. (F–G) in vitro Vδ2 T cell cytotoxic assay: (F) schematic representation of the experimental setup: PBMCs were incubated for 12 days with the indicated stimulus or inhibitors in the presence of IL‐2. The Vδ2 T cells were isolated from PBMC cultures by magnetic purification and co‐incubated with Jurkat cell at different cell to cell ratios. (G) Percentage of Jurkat cells survival after co‐incubation with Vδ2 T cells assessed by flow cytometry and normalised to Jurkat cell cultures without Vδ2 T cells (Mean ± SEM, *n* = 5); (repeated measures one‐way ANOVA followed by Tukey's multiple comparisons test, *p* value < 0.05). Created with Biorender.

Apart from cytokine production, Vδ2 T cells exert their function via cytotoxic activity [77, 78]. Therefore, we investigated whether mevalonate metabolism is also essential for the cytotoxic function of these cells. With the same in vitro experimental setup (Figures 1B and 5A), we assessed the granzyme B and perforin production by Vδ2 T cells as well as the degranulation marker CD107a [79, 80] (Figure 7). Similar to cytokine production capacity, IPP did not affect the production of these cytotoxic molecules (Figure 7A–C). Zoledronate and fluvastatin at high doses reduced CD107a expression and granzyme B production by Vδ2 T cells, while 6‐fluoromevalonate had only an effect on CD107a expression (Figure 7A–C). Furthermore, the percentage of granzyme B‐producing Vδ2 T cells decreased in both tunicamycin‐ and GGTI2133‐treated cultures (Figure 7D), resembling the phenotypes of Vδ2 T cells in zoledronate‐ and statin‐treated cultures. Tunicamycin and GGTI2133 treatment also significantly reduced the numbers of perforin‐producing Vδ2 T cells (Figure 7D). Furthermore, mevalonate pathway inhibition by a high dose of fluvastatin resulted in reduced cytotoxic molecule production by CD8 T cells (Figure S7A). We therefore postulate that the mevalonate pathway may also support granzyme B and perforin production by T cells. We next assessed whether the cytotoxic properties of Vδ2 T cells are also affected by mevalonate pathway inhibition in vivo. Consistent with our in vitro findings, hypercholesterolemia patients undergoing statin therapy displayed a reduced proportion of granzyme B‐ and perforin‐producing Vδ2 T cells (Figure 7E) but among αβ T cells, only perforin‐producing CD8 T cell numbers were decreased (Figure S7B). This data show that the mevalonate pathway is important for the production of cytotoxic molecules by Vδ2 T cells.

To determine whether these observations are of functional relevance, we established an in vitro cytotoxic assay (Figure 7F,G). To this end, we purified Vδ2 T cells from PBMC cultures treated with mevalonate pathway inhibitors and co‐incubated them with zoledronate‐pulsed Jurkat cells (Figure 7F). The survival rate of target cells was assessed by flow cytometry. The addition of Vδ2 T cells purified from RPMI alone cultures decreased the survival of Jurkat cells, confirming the cytotoxic properties of Vδ2 T cells (Figure 7G) while the survival rate of target cells upon incubation with zoledronate‐ and fluvastatin‐treated Vδ2 T cells was higher, further validating that mevalonate metabolism also plays a role in the cellular cytotoxic activity of Vδ2 T cells against cancer cells. Altogether, these data indicate that mevalonate pathway inhibition affects the cytotoxic properties of Vδ2 T cells.

## 3 Discussion

Vδ2 T cells play an important role in host defence against infection and in anti‐cancer immune surveillance. Thanks to their potent killing properties, easy expansion with aminobisphosphonate drugs and HLA‐independent activity, they have been gaining increasing attention in the field of T cell therapies [81]. Thus, several in vitro expansion protocols have been established to effectively generate pure Vδ2 T cell populations within 11–15 days in PBMC culture before transferring to patients [14, 40, 41, 42, 43, 44, 45]. Yet, the preliminary trials with zoledronate‐expanded Vδ2 T cells in cancer patients showed limited effectiveness [16, 17]. Our study is the first one to compare phosphoantigen‐expanded with aminobisphosphonate‐expanded Vδ2 T cells and revealed that, despite robust proliferation, zoledronate impacted Vδ2 T cells and reduced their pro‐inflammatory activity, either by a direct effect on Vδ2 T cells or by an indirect effect on other cells in the PBMC cultures, up to 21 days in the culture. Due to its ability to inhibit FPP synthase, zoledronate is known to cause the accumulation of IPP in antigen presenting cells and consequently activate Vδ2 T cells in the context of butyrophilins [10, 82, 83, 84]. Consistently, we found numerous commonalities in the transcriptional programs and kinase activities of IPP‐ and zoledronate‐expanded Vδ2 T cells. For example, transcriptome analysis revealed that pathways related to leukocyte activation, cell cycle regulation and DNA replication are up‐regulated in IPP‐ and zoledronate‐treated Vδ2 T cells, while kinome analysis revealed that the activity of kinases related to proliferation, differentiation and cell cycle regulation was highly increased in IPP‐ and zoledronate‐treated cells. These observations are in line with the high proliferation rate of the cells in these conditions. Similarly, the activity of kinases downstream of TCR signalling, such as LCK, SCR, ZAP70, was also upregulated, providing further evidence that IPP can potently activate the TCRVδ2. Among kinases whose activity was downregulated in Vδ2 T cells by zoledronate but not IPP, we found MAPKs downstream of the GTPases such as MAP3K5 and its interaction partner MAP3K6, which activate the JNK and P38 kinase signalling pathways [85]; MAP2K4, a key component of the P38 pathway that plays an essential role in T cell proliferation and cytokine production, including IFN‐γ [86, 87, 88]; and MAP2K7, which is involved in the pro‐inflammatory cytokine response in mouse macrophages [89]. These show that JNK and P38 signalling may be hindered in Vδ2 T cells upon zoledronate treatment, which is in line with our Western Blot results showing an accumulation of the unprenylated form of their activating small GTPases: RAC and RHOA, respectively, in the cytosol. Based on the reported importance of RAC proteins and the downstream JNK and P38 signalling pathways for the induction of IFN‐γ expression in CD4 and CD8 T cells [87, 88, 90], we hypothesise that these signalling pathways are also required for the cytokine production by γδ T cells. Due to the fact that JNK and P38 kinases activate the Activator Protein 1 (AP‐1) transcription factor composed of Fos and Jun proteins [91, 92], both *TNF* and *IFNG* gene regulatory elements possess Jun and Fos binding sites [93, 94] and their expression is regulated by these transcription factors in conventional T cells [95, 96, 97], we hypothesise that the zoledronate‐impaired signalling in Vδ2 T cells might act via compromised AP‐1 activity. In previous studies, osteoporosis and cancer patients who were prescribed aminobisphosphonates showed a selective reduction of Vδ2 T cells [98] and progressive clinical deterioration [99], respectively, indicating that zoledronate may play a role in dampening Vδ2 T cell functions in vivo. Yet, these speculations require further examination by in vitro assays and clinical studies.

However, inconsistent with the flow cytometry results, TNF transcript levels are up‐regulated in zoledronate‐treated Vδ2 T cells. One reason for this discrepancy might be due to the fact that RNA‐seq analysis was performed on expanded but not restimulated cells, in contrast to the cytokine production assessment by flow cytometry and kinome assessment by PamGene, where cells were restimulated for 4 h or 10 min prior to intracellular staining and phosphorylation assay, respectively. Although restimulation with phosphoantigens is the most physiological way to activate human Vδ2 T cells, in our experiments we used the anti‐CD3 and anti‐CD28 antibodies [98] because: (1) it allowed us to address the Vδ2 T cell function independently of the butyrophilin presence in the culture, (2) it allowed us to simultaneously assess cytokine production by CD4 and CD8 T cells in the culture, (3) it is more physiological stimulation than that with PMA/ionomycin which bypasses the TCR signalling. Although not all Vδ2 T cells express CD28 [100], we did not observe differences in cytokine production capacity by Vδ2 T cells in our cultures upon anti‐CD3 restimulation alone and the combination of anti‐CD3 and anti‐CD28 (data not shown).

The transcriptome analysis further revealed an upregulation of the TIM3 transcript in zoledronate‐treated Vδ2 T cells, which was consistent with the protein levels assessed by flow cytometry. Furthermore, we observed a slight increase in the expression of the TIM3 ligands CD66a/c/e in zoledronate‐treated cultures. TIM3 is a well‐known immune‐suppressive checkpoint receptor [101], which intervenes with proximal TCR signalling [75, 76]. However, our kinome analysis did not reveal differences in LCK, SCR or ZAP70 activity in zoledronate‐ versus IPP‐treated cells. These observations urge further investigation and suggest that upregulation of TIM3, but not other checkpoint markers, may be partially responsible for the attenuated Vδ2 T cell function in zoledronate‐treated cultures. This is in line with recent findings revealing that blocking PD‐1 and CTLA4 receptors with antibodies does not improve the cytokine expression of zoledronate‐expanded human γδ T cells [102]. Yet, our observations support the use of a combination therapy blocking PD‐1 and TIM3 signalling for a better outcome in cancer patients [103]. As we have not investigated the activation‐induced anergy of T cells in our study, it could contribute the zoledronate‐induced phenotype of Vδ2 T cells and should be a subject of further investigations. Although our results on purified Vδ2 T cells suggest a direct effect of zoledronate on the cytokine production capacity, we cannot exclude the indirect effect mediated by other cellular components of the PBMCs due to the limitation of purification technique in the study. Generation of Vδ2 T cell clones would be useful to clarify these mechanisms.

Moreover, we found that other mevalonate pathway inhibitors: statins and 6‐fluoromevalonate, while having a distinct effect on proliferative capacity, also compromised TNF and IFN‐γ cytokine production and cytotoxic activity of Vδ2 T cells. In vitro inhibition of the mevalonate pathway using 6‐fluoromevalonate did not result in any significant changes in Vδ2 T cell numbers, in line with the phenotype of hyper‐IgD syndrome patients. This is likely due to the lack of IPP accumulation upon 6‐fluoromevalonate treatment. Yet, Vδ2 T cells from hyper‐IgD syndrome patients and from the 6‐fluoromevalonate‐treated PBMC cultures showed reduced TNF‐ and IFN‐γ production capacity. 6‐fluoromevalonate treatment further resulted in upregulation of checkpoint receptors TIM3 and LAG3 without affecting the expression of their ligands. These results advocate the assessment of the exhaustion status of T cells in Hyper‐IgD syndrome patients to verify whether this contributes to the pathology of the syndrome.

We further observed that a high concentration of statins significantly decreased the number of Vδ2 T cells and the production of effector molecules: TNF, IFN‐γ and granzyme B as well as the degranulation marker CD107α. Similarly, patients undergoing statin treatment had reduced numbers of cytokine‐producing Vδ2 T cells, even though the concentration of statin in peripheral blood is likely much lower than that used in in vitro experiments [104]. This could be due to the toxicity of the drug, which was previously reported on CD4 T cells [105]. However, mevalonic acid restored the viability and cytokine production of Vδ2 T cells in fluvastatin‐treated PBMC cultures, indicating that mevalonate deficiency, but not other processes, is responsible for the reduced viability and function of fluvastatin‐treated Vδ2 T cells. Although little has been reported on the effect of statins on Vδ2 T cells, our observations are consistent with previous studies showing impaired proliferation capacity and IFN‐γ production by conventional T cells in mice and humans [105, 106].

Our RNA‐seq analysis revealed that many transcripts related to negative regulation of immune cell functions are upregulated by statin treatment, while numerous genes related to cell division as well as glucose metabolism are downregulated in fluvastatin‐treated Vδ2 T cells. However, energy metabolism profiling by SCENITH [64] did not confirm a decrease in glycolytic capacity of Vδ2 T cells in statin‐treated PBMC cultures. It is known that activation of T cells as well as IFN‐γ production by mouse γδ T cells highly depend on energy derived from aerobic glycolysis [107, 108, 109]. Furthermore, the activities of many kinases related to TCR activation and signalling as well as many proliferation‐related tyrosine receptor kinases such as TYRO3 and MER responsible for AKT and ERK signalling [110] were significantly downregulated in fluvastatin‐treated Vδ2 T cells, consistent with the decreased proliferative activity of the cells. Statin also compromised the activity of kinases downstream of GTPases such as JNK1,2,3, P38δ and ERK1,2. The reduced activity of these signal transduction pathways may be attributed to both decrease in receptor signalling and the abrogation of small GTPase prenylation. Although no mutual kinases downstream of small GTPases were downregulated in zoledronate‐ and fluvastatin‐treated Vδ2 T cells, one commonly downregulated phosphosite was found on the BTLN protein, which regulates lymphocyte proliferation and cytokine production [111, 112]. It is therefore of interest to investigate the role of BTLN in Vδ2 T cells further. We also found very few commonalities in the transcriptional programs of zoledronate‐ and fluvastatin‐treated cells. Among the commonly upregulated transcripts were genes related to leukocyte‐mediated immunity such as CD137 and CX3C motif chemokine receptor 1 (CX3CR1) as well as poorly characterised chromosome condensation 1‐like (CHC1L), whereas the commonly downregulated gene was protein‐glucosylgalactosylhydroxylisine glucosidase (PGGHG) which removes glucose residue from collagen‐like proteins [113]. While CD137 is known to be expressed on activated T cells and contribute to their survival and effector function [114], CX3CR was found on differentiated CD8 T cells with cytotoxic activity [115, 116], the role of RCBTB2 and PGGHG in the immune system is not known. All these genes might be involved in impaired Vδ2 T cell function upon mevalonate pathway inhibition and urge follow‐up investigation. We conclude that compromised intracellular signalling in fluvastatin‐treated Vδ2 T cells results in weakened effector function, which manifests in decreased production of pro‐inflammatory cytokines. We show that although all mevalonate pathway inhibitors used in the study suppress TNF and IFN‐γ production, they distinctly affect biological processes in Vδ2 T cells.

Although TNF and IFN‐γ expression is impaired upon mevalonate inhibition, our ATAC‐seq analysis suggested no significant alterations in the chromatin accessibility at the *TNF* and *IFNG* loci upon fluvastatin and zoledronate treatment. Analysis of DNA methylation and histone modifications might provide better insights into epigenetic events taking place in Vδ2 T cells upon mevalonate pathway inhibition. For example, based on animal models and in vitro studies, it has been postulated that statins affect histone acetylation, while cohort studies suggest that statin therapy affects DNA methylation in blood cells [117, 118, 119]. Furthermore, the use of histone deacetylase inhibitors has been shown to affect the effector functions of Vδ2 T cells [120], suggesting indeed the important role of epigenetic mechanisms in shaping Vδ2 T cell responses.

Among the biological processes examined downstream of the mevalonate pathway, we found that protein prenylation regulates both TNF and IFN‐γ production by Vδ2 T cells. Geranylgeranylation inhibitors caused a significant decrease in both TNF and IFN‐γ production by Vδ2 T cells, while inhibition of farnesylation has a milder effect. Consistently, we found an accumulation of unprenylated small G proteins, namely RAC, RHOA, RAP1 and RAS in the cytosol of Vδ2 T cells isolated from zoledronate‐treated PBMC cultures. We also observed an accumulation of unprenylated GTPases in fluvastatin‐ and IPP‐treated cells, although this was not significant. While the depletion of RAS, RHO and RAB from the lipid rafts upon simvastatin treatment results in impaired intracellular signalling [121], the effect of IPP on protein prenylation requires further investigation. Of note, IPP stimulation might induce a global increase in small GTPase levels, including the prenylated form. Our assessment did not allow us to look at the prenylated protein in the membrane fraction and the ratio of unprenylated vs. prenylated forms due to technical limitations such as the scarce number of Vδ2 T cells obtained from RPMI and statin‐treated cultures, yet the evaluation was based on previous reports [122].

Cholesterol synthesis is another metabolic process fueled by the mevalonate pathway. Cholesterol is essential for stabilising lipid raft structure and amending TCR signalling [123, 124, 125]. Furthermore, its role in αβ T cell dysfunction and exhaustion has also been postulated [126, 127]. Therefore, a decrease in cholesterol levels in T cells may affect effector signalling, causing the arrest of proinflammatory effector action such as cytokine release. A previous study showed that statins and zaragozic acid induce opposite effects on IFN‐γ production by T cells [122]. In our study, blocking cholesterol synthesis by zaragozic acid did not cause a decrease in cytokine production, and fluvastatin treatment did not result in the depletion of cellular cholesterol. This observation is in line with previous studies on human T cells, where authors postulate no effect of statins on cholesterol levels and lipid rafts [121, 128]. Indeed, T cells can maintain cellular cholesterol pools through extracellular uptake [129, 130]. Therefore, cellular cholesterol levels will only be affected if extracellular cholesterol is depleted, which is not the case in our PBMC cultures. Thus, it is unlikely that zoledronate, fluvastatin, and 6‐fluoromevalonate affect TCR signalling through cholesterol depletion. Although cholesterol synthesis is not responsible for the phenotype we observed, it most likely still plays a role in Vδ2 T cell function. Further investigation with potent cholesterol‐depleting agents, such as methyl‐β‐cyclodextrin and in lipid‐free medium, would unravel the function of cholesterol in Vδ2 T cell biology.

In addition to impaired cytokine production, the cytotoxic activity of zoledronate‐ and fluvastatin‐treated Vδ2 T cells towards cancer cells was also reduced. Our data revealed that the expression of cytotoxic molecules: granzyme B and perforin [7] was decreased in Vδ2 T cells in patients undergoing statin therapy. Granzyme B expression was also decreased by zoledronate and fluvastatin treatments in PBMC cultures. These results are consistent with a recent study where authors showed that the ability of in vitro expanded Vδ2 T cells to kill kidney carcinoma cells is restricted by granzyme B production and, to a lesser extent, by perforin [131]. Furthermore, we found that N‐glycosylation and protein geranylgeranylation are important processes for cytotoxic granule production by Vδ2 T cells. These findings are in line with reports revealing that granzymes and perforin undergo N‐glycosylation [132, 133]. Altogether, inhibition of the mevalonate pathway results in reduced cytotoxic function in Vδ2 T cells.

Thus, our data suggest that the two processes: effector molecule production and proliferation are uncoupled in Vδ2 T cells. In line with this hypothesis, an earlier study by Ryan et al. postulated a functional heterogeneity of the Vδ2 T cell compartment where the proliferative and effector capacities were mutually exclusive [7]. Moreover, Vδ2 T cell function seems to be more sensitive to the modulation of the mevalonate pathway than the function of conventional T cells.

Taken together, our study revealed the importance of mevalonate metabolism for the proper effector function of Vδ2 T cells. Although all mevalonate pathway inhibitors used in the study resulted in compromised Vδ2 T cell function, the underlying molecular mechanisms of their action are different. While we focused our analysis on cholesterol synthesis, protein N‐glycosylation and protein prenylation, other processes downstream of mevalonate metabolism, such as ubiquinone synthesis, might be affected by the inhibitors and contribute to the phenotype. Given the wide usage of mevalonate pathway inhibitors in clinics, our observations are vital for improving patient care and the effectiveness of treatment for the deadliest diseases: cardiovascular disease and cancer.

## 4 Methods

### 4.1 Cell Lines and Tissue Culture

Jurkat cell line was a kind gift from Prof. Dr. Dietmar Schmucker at the University of Bonn. It was subjected to mycoplasma testing prior to experiments. It was maintained in Roswell Park Memorial Institute (RPMI) 1640 supplemented with 10% fetal bovine serum (FBS; Pan Biotech, Aidenbach, Germany) and 1% glutamax (Gibco, Grand Island, USA).

### 4.2 Human Study Oversight

Buffycoats from healthy donors were obtained from Uniklinikum Bonn following approval of the Ethics Committee for Clinical Trials on Humans and Epidemiological Research with Personal Data of the Medical Faculty of the Rheinische Friedrich‐Wilhelms‐Universität Bonn (approval numbers 148/20 and 249/22). Informed Consent was obtained from all participants. Human peripheral blood mononuclear cells (PBMCs) were isolated and reconstituted in RPMI medium (Pan Biotech) supplemented with 10% FBS (Pan Biotech), 1% sodium pyruvate (Gibco), 1% glutamax (Gibco) and 0.1% gentamicin (Gibco). All cells were maintained at 37°C in a 5% CO_2_ humidified incubator.

### 4.3 Clinical Study

Hypercholesterolemia patients were recruited as part of the clinical study approved by the Arnhem‐Nijmegen Ethical Committee (no. NL72155.091.20, CMO 2020‐6543) in Radboud University Medical Center, Nijmegen, the Netherlands, according to the Declaration of Helsinki and Good Clinical Practice. Informed Consent was obtained from all participants. The demographic data of participants are presented in Table S1. All patients did not use cholesterol‐lowering drugs at inclusion in the study. They were subsequently treated with statins at the discretion of the treating physician (Table S1).

### 4.4 Clinical Hyper Inflammatory Disease Study

Patients with Hyper IgD syndrome were recruited as part of a clinical study approved by the Arnhem‐Nijmegen Ethical Committee (no. NL32357.091.0 and NL42561.091.12) in Radboud University Medical Center, Nijmegen, the Netherlands, according to the Declaration of Helsinki and Good Clinical Practice. Informed Consent was obtained from all participants. The demographic data of participants are presented in Table S2.

### 4.5 PBMCs Isolation

Freshly collected buffy coats from healthy volunteers were transferred to a 200 mL culture flask. The blood was diluted 1:4 with phosphate‐buffered saline (PBS) buffer. The diluted blood was layered over Pancoll Human (Pan Biotech) and density gradient centrifuged (610 cf., 30 min, 22°C) to separate human peripheral blood mononuclear cells (PBMCs) from red blood cells and granulocytes. The interphase rings containing the PBMCs were collected into new tubes and washed twice with cold PBS. The PBMCs were pelleted by centrifugation (1700 RPM, 15 min, 4°C), resolved and counted using trypan blue exclusion of dead cells on ahemacytometer.

### 4.6 In Vitro Inhibition of Mevalonate Pathway

#### 4.6.1 PBMC Cultures

PBMCs reconstituted as above were incubated in RPMI alone or in the presence or absence of IPP (10 μM; Sigma‐Aldrich, St. Louis, MO, US), fluvastatin (0.5 μM, 1 μM, 5 μM, 10 μM; Sigma‐Aldrich), atorvastatin (0.5, 1, 5 and 10 μM; Sigma‐Aldrich), zoledronate (1 and 5 μM; Merck, Darmstadt, Germany) or 6‐fluoromevalonate (50 and 100 μM; Sigma‐Aldrich) in a 96‐well plate at 37°C and 5% CO_2_ for 12, 14, 18 and 21 days. All cultures were supplemented with IL‐2 (100U; Peprotech, Cranbury, NJ, USA). On the designated day, the PBMC cultures were stimulated with soluble anti‐CD3 (1 μg/mL, HIT3A; BD Bioscience, Franklin Lakes, NJ, USA), anti‐CD28 (1 μg/mL; CD28.2 BD Bioscience) and were incubated with Golgi Plug (1 μg/mL; BD Bioscience) at 37°C and 5% CO_2_ for 4 h. The cultures were harvested for surface marker and intracellular staining and analysed by flow cytometry.

### 4.7 Purified Vδ2 T Cell Cultures

Vδ2 T cells were purified from the PBMC cultures by positive magnetic selection (Stemcell EasySep Release APC positive magnetic selection kit) according to the manufacturer's instructions. Purified Vδ2 T cells were incubated with RPMI alone or zoledronate (1 μM; Merck, Darmstadt, Germany) in the presence of IL‐2 (100 U; Peprotech, Cranbury, NJ, US) in 96‐well plates at 37°C and 5% CO_2_ overnight, 2, and 6 days. PBMC cultures were stimulated with soluble anti‐CD3 (1 μg/mL, HIT3A; BD Bioscience, Franklin Lakes, NJ, US), anti‐CD28 (1 μg/mL; CD28.2 BD Bioscience) and incubated with Golgi Plug (1 μg/mL; BD Bioscience) at 37°C and 5% CO_2_ for 4 h. The cultures were harvested for surface marker and intracellular staining and analysed by flow cytometry.

### 4.8 In Vitro Inhibition of Mevalonate Pathway During Cytokine Assay

PBMCs (5 × 10^6^ cells/well) were expanded by IPP (10 μM; Sigma‐Aldrich) and IL‐2 (100 U; Peprotech) in 6‐well plates for 12 days. Then, cells were transferred to 96‐well plates (0.5 × 10^6^ cells/well) and incubated with RPMI alone, fluvastatin (1 and 5 μM; Sigma‐Aldrich) or zoledronate (1 and 5 μM; Merck, Darmstadt, Germany) and incubated together with soluble anti‐CD3 (1 μg/mL; HIT3A BD Bioscience), anti‐CD28 (1 μg/mL; CD28.2 BD Bioscience) and Golgi Plug (1 μg/mL; BD Bioscience) for 4 h. The cultures were harvested for surface marker and intracellular staining and analysed by flow cytometry.

### 4.9 In Vitro Rescue of Mevalonate Pathway Inhibition

PBMCs reconstituted as above were treated with the stimuli and mevalonate pathway inhibitors as described above and supplemented with mevalonic acid (50 and 100 μM; Sigma‐Aldrich) at 37°C and 5% CO_2_ for 12 days. On day 12, the PBMC cultures were stimulated with soluble anti‐CD3 (1 μg/mL; HIT3A BD Bioscience), anti‐CD28 (1 μg/mL; CD28.2 BD Bioscience) and incubated with Golgi Plug (1 μg/mL; BD Bioscience) at 37°C and 5% CO_2_ for 4 h. The cultures were harvested for surface markers and intracellular staining and analysed by flow cytometry.

### 4.10 Clinical Study of Hypercholesterolemia Patients

The peripheral blood from hypercholesterolemia patients was collected at baseline before statin treatment and after 3 months of statin treatment, as well as from healthy volunteers. PBMCs were isolated and reconstituted as described above and then stimulated with soluble anti‐CD3 (1 μg/mL; HIT3A BD Bioscience), anti‐CD28 (1 μg/mL; CD28.2 BD Bioscience) and incubated with Golgi Plug (1 μg/mL; BD Bioscience) at 37°C and 5% CO_2_ for 4 h. The cultures were harvested for surface markers and intracellular staining and analysed by flow cytometry.

### 4.11 Clinical Study of Hyper IgD Syndrome Patients

The peripheral blood of Hyper IgD syndrome patients and healthy volunteers was collected at baseline, PBMCs were isolated and reconstituted as described above. The PBMCs were then stimulated with soluble anti‐CD3 (1 μg/mL; HIT3A BD Bioscience), anti‐CD28 (1 μg/mL; CD28.2 BD Bioscience) and incubated with Golgi Plug (1 μg/mL; BD Bioscience) at 37°C and 5% CO_2_ for 4 h. The cultures were harvested for surface marker and intracellular staining and analysed by flow cytometry.

### 4.12 In Vitro Inhibition of Processes Downstream Mevalonate Pathway

PBMCs (5 × 10^6^ cells/well) were expanded by IPP (10 μM; Sigma‐Aldrich) and IL‐2 (100 U; Peprotech) in a 6‐well plate for 12 days. Then, PBMCs were transferred to a 96‐well plate (0.5 × 10^6^ cells/well) and incubated with RPMI alone, zaragonzic acid (75 μM, 100 μM; Sigma‐Aldrich), tunicamycin (1 and 5 μM; Sigma‐Aldrich), geranylgeranyltransferase inhibitor 2133 (GGTI2133; 5, 10, 30 and 50 μM; Sigma‐Aldrich), geranylgeranyltransferase inhibitor 286 (GGTI286; 30, 50, 75 and 100 μM; Sigma‐Aldrich), geranylgeranyltransferase inhibitor 298 (GGTI298; 5, 10, 30 and 50 μM; Sigma‐Aldrich), and farnesyltransferase inhibitor 277 (FTI 277; 50, 100 and 150 μM; Sigma‐Aldrich) overnight. The PBMC cultures were stimulated with soluble anti‐CD3 (1 μg/mL; HIT3A BD Bioscience), anti‐CD28 (1 μg/mL; CD28.2 BD Bioscience) and incubated with Golgi Plug (1 μg/mL; BD Bioscience) for 4 h. The cultures were harvested for surface marker and intracellular staining and analysed by flow cytometry.

### 4.13 Surface Marker and Intracellular Staining by Flow Cytometry

PBMCs were incubated with the following antibodies against cell surface markers: anti‐human CD3 Pacific blue (UCHT1; BioLegend, San Diego, CA, US), anti‐human CD45 Brilliant Violet 605 (HI30; BioLegend), anti‐human CD45 PE/Dazzle 594 (HI30; BioLegend), anti‐human CD4 PE‐Cy7 (OKT4; BioLegend), CD4 Brilliant Violet 605 (OKT4; BioLegend), anti‐human PD‐1 PerCP 5.5 (A17188B; BD Bioscience), anti‐human CD8 APC‐Cy7 (SK1; BioLegend), anti‐human LAG3 Dazzle red (11C3C65; BioLegend), anti‐human TIM3 PerCP‐Cy5.5 (F38‐2E2; BioLegend), anti‐human CTLA4 Brilliant Violet 605 (BNI3; BioLegend), anti‐human TCR Vδ2 FITC (REA711; Miltenyi Biotec, Bergisch Gladbach), anti‐human TCR Vδ2 APC (REA711; Miltenyi Biotec), anti‐human CD80 PE (2D10; BioLegend), anti‐human CD86 FITC (BU63; BioLegend), anti‐human CD66a (CEACAM1) PE (ASL‐32; BioLegend), anti‐human CD274 (PDL1) FITC (MIH2; BioLegend), anti‐human CD107a PE (H4A3; BioLegend), Live‐or‐Dye Fixable stain (Biotium, Fremont, CA, US) and Live/dead Fixable Aqua Dead stain (Invitrogen, Waltham) at 4°C in the dark for 30 min. The cells were washed with PBS then permeabilized/fixed in Cytofix permeabilization/fixation reagent (BD biosciences) for 30 min. The cells were washed with Cytofix permeabilization/washing buffer (BD biosciences) twice and incubated in Cytofix permeabilization/washing buffer with the following antibodies against intracellular markers: anti‐human TNFα APC (MAb11; BioLegend), anti‐human IFNγ PerCP‐Cy5.5 (B27; BD Pharmingen), anti‐human granzymeB Alexa Fluor 687 (BG11; BioLegend), anti‐human Perforin PE‐Cy7 (dG9; BioLegend) anti‐human CD3 Pacific blue (UCHT1; BioLegend) and anti‐human puromycin AF488 (12D10; Millipore) at 4°C in the dark for 30 min. The cells were washed with PBS then fixed in CellFIX reagent (BD Bioscience) and stored at 4°C in the dark. Colour compensation was done using OneComp eBeads (BD Bioscience). FACS analysis was performed on LSR II (BD Bioscience). Data analysis is performed using FlowJo vX.07 software.

### 4.14 RNA Sequencing Sample Preparation

0.5 × 10^6^ Vδ2 T cells (CD45^+^ CD3^+^ TCR Vδ2^+^) in each culturing condition from the corresponding donor were sorted from PBMC cultures on BD Aria3 flow cytometer and stored in QIAzol (Qiagen, Hilden, Germany). Libraries were assembled by adapting the SMART‐Seq2 protocol [134]. Qubit dsDNA HS Assay kit (Thermo Fisher Scientific, Waltham, MA, US) was applied for library quantification. Library size estimation was performed on the TapeStation 4200 High‐sensitivity D500 assay system (Agilent Technologies, Santa Clara, CA, US). Pooled samples were then sequenced on a NextSeq500 using High Output v.2 chemistry (SR 75 bp). Raw data was collected as FASTQ files and demultiplexed with bcl2fastq2 v2.20.

### 4.15 RNA‐Seq Data Analysis

Pseudo‐alignment was performed with Kallisto v.0.440 and mapped against the GRCh38p13 human reference genome (GENCODE v.27). Raw counts were imported, and low‐count genes were excluded by pre‐filtering (> 10 reads in at least 3 samples and only protein coding transcripts) resulting in 13 084 transcripts. DEseq2 pipeline was applied to normalise the count and rlog transform the data using default parameters [135]. Surrogate variable analysis (SVA) was used to identify latent variables (3 significant SVs) that caused the batch effects and the variables were included in the DEseq2 model. Transformed counts were corrected for 3 SVAs using the function provided in the limma R package. All the present transcripts were submitted as input for principal component analysis (PCA). Standard differential expression analysis was performed for IPP‐, zol‐ and statin‐treated vs. control comparisons using adjusted *p* value threshold equal to 0.05 and a foldchange cut‐off of 1.5. IHW was used as multiple testing parameter and log2 fold change shrinkage was applied. Hierarchical clustering of variable transcripts within the dataset were presented in the heatmap and the selected differentially expressed (DE) genes were annotated. The results from the differential genes expression analysis were visualizedas volcano plots. Gene ontology (GO) enrichment analysis was performed for DE transcripts in the respective comparison using gene ontology set of biological processes using the R tool clusterprofiler. The count and *p* value (*p* < 0.05) associated with each GO term were depicted in enrichment dotplot.

For co‐expression network analysis, all present and batch‐corrected transcripts were used as input. The inflection point was calculated, and 7184 top variable genes were included as input for hCoCena (horizontal construction of co‐expression networks and analysis). Pairwise Pearson's correlation coefficients were calculated using the R package Hmisc (v4.1–1) to identify gene pairs whose expression patterns are positively correlated across all tested samples. A Pearson correlation coefficient cutoff of 0.912 (6023 nodes and 63 226 edges) based on the balance of scale‐free topology, number of graph components and edges was selected to construct an undirected co‐expression network. Then, the “leiden” algorithm in igraph (v1.2.1) [the igraph software package for complex network research] was applied to perform unbiased clustering and this step was repeated 10 times. Modules with less than 40 genes were discarded. For each gene, the group fold changes (GFCs) for RPMI‐, IPP‐, zol‐ and statin‐treated conditions were computed by calculating the average expression of that gene across all samples and determining the fold change of the gene's mean expression within each condition relative to the overall mean expression. Then, the hclust function (cluster package, version 2.1.0) was used to perform agglomerative hierarchical clustering. The clinical parameters and conditions clustered by their GFCs were presented in a heatmap. All clusters individually were used for GO and Hallmark enrichment analyses. The results were filtered for the five most significant terms with adjusted *p* values ≤ 0.05 per cluster. If clusters are missing in the plot, then no significantly enriched terms were found.

### 4.16 ATAC‐Sequencing Sample Preparation

0.1 × 10^6^ Vδ2 T cells sorted on BD Aria3 flow cytometer as for the RNA‐seq analysis. The cells were lysed in lysis buffer (10 mM Tris–HCl, pH 7.4, 10 mM NaCl, 3 mM MgCl_2_, 0.1% IGEPAL CA‐630) and processed for nuclei isolation. Transposition reaction following the protocol of Buenrostro et al. [136] was performed using in‐house Tn5 and DMF‐Tn5 buffer. The transposed DNA was purified by MinElute Purification Kit (Qiagen) and amplified by PCR. Library quantification was performed using a Qubit dsDNA HS Assay Kit (Thermo Fisher Scientific). Library size estimation was performed on the TapeStation 4200 High‐sensitivity D500 assay system (Agilent Technologies). Paired‐end Assay for Transposase‐Accessible Chromatin using Sequencing (ATAC‐Seq) was performed on pooled samples at NovaSeq SP flow cell with v.1 chemistry (PE 75 bp).

### 4.17 ATAC‐Sequencing Analysis

ATAC‐Seq data preprocessing was based on the guideline on Galaxy training network [137, 138]. First, the FASTQ files were demultiplexed with bcl2fastq2 v2. The adapters were trimmed using Cutadapt (Martin, 2011). Then, the sequencing reads were mapped using bowtie2 v 2.3.5 against the GRCh38p13 human reference genome (Langmead and Salzberg, 2012). Then, duplicated reads were removed using the Picard MarkDuplicates function. Bam files were exported and visualised by Integrative Genomic Viewer (IGV) [139].

### 4.18 SCENITH

PBMCs were plated at 0.3 × 106 cells/well in 96‐well plates at 37°C and 5% CO_2_ for 12 days. On day 12, the cells were incubated with 2‐deoxy‐D‐Glucose (100 mM; Sigma‐Aldrich), Oligomycin (10 μM; Sigma‐Aldrich) and a combination of 2‐Deoxy‐D‐Glucose and Oligomycin for 30 min at 37°C. Puromycin (10 mg/mL; Sigma‐Aldrich) was added to the culture and incubated for 45 min at 37°C. Cells were washed in cold PBS and harvested for surface marker and intracellular staining and analysed by flow cytometry. The metabolic dependencies and capacities were calculated following the published method [64].

### 4.19 Western Blot Sample Preparation and Image Analysis

PBMCs (5 × 10^6^ cells/well) were incubated with RPMI alone, IPP (10 μM; Sigma‐Aldrich), fluvastatin (1 μM; Sigma‐Aldrich) or zoledronate (1 μM; Sigma‐Aldrich) in 6‐well plates for 12 days in the presence of IL‐2 (100 U; Peprotech). 0.5 × 10^6^ Vδ2 T cells were purified from the PBMC cultures by the Stemcell EasySep Release APC positive magnetic selection kit (Stemcell technologies, Vancouver, Canada) according to the manufacturer's instructions and stored as pellets. The pellets were resuspended and lysed by RIPA buffer containing protease and phosphatase inhibitors (Thermo Fisher Scientific) for 30 min at 4°C. The lysates were then centrifuged at 14000 g at 4°C for 10 min. The supernatant containing the cytosolic fraction of the protein lysate was collected. Pierce BCA protein quantification assay was performed to determine the concentration of protein in the lysate (Thermo Fisher Scientific). Lysate was then boiled together with Laemmli sample buffer (Bio‐rad, Hercules, CA, US) for 5 min at 95°C and proceeded with SDS‐PAGE. Equal amounts of protein lysate (10 μg) were separated on a 10% Mini‐PROTEAN TBE‐Urea Gel (Bio‐rad) and transferred to a Trans‐Blot Turbo Mini 0.2 μm Nitrocellulose membrane (Bio‐rad) using the Trans‐Blot Turbo Transfer System (Bio‐rad). The blot was blocked in 5% non‐fat dried milk (Carl Roth, Karlsruhe, Germany) for 1 h prior to overnight incubation with the following primary antibodies: anti‐RAC 1/2/3 (Cell signalling, Danvers, MA, US), anti‐RHOA (Cell signalling), anti‐Pan RAS (Santa Cruz Biotechnology), anti‐RAP1A/RAP1B (Cell signalling), anti‐b‐actin (Biolegend), anti‐phospho p44/42 MAPK (Erk1/2; Cell signalling), anti‐P44/42 MAPK (Erk1/2; Cell signalling). Blot was washed 3 times with Tris Buffered Saline‐Tween (TBS‐T) and incubated with anti‐mouse IgG (Cell signalling) or anti‐rabbit IgG HRP‐conjugated secondary antibodies (Cell signalling). The blot was incubated with Pierce ECL Western Blotting Substrate (Thermo Fisher Scientific) after 3 washes with TBS‐T and analysed by ChemiDoc imager (Bio‐rad).

### 4.20 PamGene Sample Preparation and Analysis

PBMCs were isolated from buffy coats of healthy human donors. The PBMC cultures (5 × 10^6^ cells/well) were incubated with RPMI alone, IPP (10 μM; Sigma‐Aldrich), fluvastatin (1 μM; Sigma‐Aldrich) or zoledronate (1 μM; Sigma‐Aldrich) in 6‐well plates for 12 days in the presence of IL‐2 (100 U; Peprotech). Vδ2 T cells were purified from the PBMC cultures by the Stemcell EasySep Release APC positive magnetic selection kit (Stemcell technologies). The purified Vδ2 T cells were stimulated with soluble anti‐CD3 (1 μg/mL; HIT3A BD Bioscience) and anti‐CD28 (1 μg/mL; CD28.2 BD Bioscience) for 10 min and immediately lysed by M‐PER Mammalian Extraction Buffer (Thermo Fisher Scientific) containing Halt Phosphatase and Protease Inhibitor cocktail (Thermo Fisher Scientific). The extracted protein was quantified by the Pierce BCA protein quantification assay (Thermo Fisher Scientific). The kinase activity of the protein lysate was determined on PTK PamChip and STK PamChip arrays (Pamgene International BV, Wolvenhoek, Netherland) and performed on a PamStation according to the manufacturer instructions. Phosphorylation signals captured from hundreds of phosphosites on the array by the PamStation were quantified using BioNavigator software 6.1 (PamGene International BV). Phosphosites that show a significant difference compared to control (*p* < 0.05) were further analysed. Differentially active kinases were identified by the Upstream Kinase Analysis (UKA) algorithm specifying the kinase‐phosphosite relationship (Median Final score > 1.2). The result is depicted using the Upstream Kinase Tool such as the CORAL kinome tree based on the kinase functional class scoring [140, 141].

### 4.21 In Vitro Cytotoxic Assay

Jurkat cell line cultures (~3 × 10^6^ cells/mL) were pulsed with zoledronate (10 μM; Sigma‐Aldrich) in a 10 mL flask overnight prior to the co‐culture with Vδ2 T cells. The cells were centrifuged at 1500 RPM at 4°C for 5 min and washed with their corresponding growth media two times. The pellets were then resuspended in RPMI containing 0.02% gentamycin.

Vδ2 T cells were purified from the PBMC cultures by positive magnetic selection (Stemcell EasySep Release APC positive magnetic selection kit) according to the manufacturer's instructions. Effector Vδ2 T cells were co‐incubated with the target cells at the effector cell: target cell ratio of 4:1, 2:1, 1:1, 1:2, 1:4 and 1:8 overnight. The cultures were then analysed by flow cytometry for live and dead cells and surface markers expression as described above.

### 4.22 Statistics

Acquired data from in vitro study was analysed statistically in case of paired non‐parametric comparison: repeated measures one‐way ANOVA followed by Tukey's multiple comparisons test and Wilcoxon test; and unpaired non‐parametric comparison: Mann–Whitney test was applied. All statistical analyses were performed on graphic analysis software GraphPad Prism 8.4 (GraphPad Software Inc.). Result considered as significant with *p* value < 0.05 (*), < 0.01 (**), < 0.001 (***) or < 0.0001 (****). Western blot protein quantification analysis was done using imageJ.

## Author Contributions

Conceptualization: K.P. Methodology: T.K.S., B.A., J.B. and K.P. Investigation: T.K.S., B.A. and K.P. Data curation: T.K.S., B.A., N.R. and K.P. Formal analysis: T.K.S., B.A., N.R. and K.P. Resources: H.B., S.B., N.R., L.A.B.J., J.L.S., M.G.N. and K.P. Writing – original draft preparation. T.K.S. and K.P. Writing – review and editing: K.P., M.G.N, L.A.B.J., J.L.S., N.R., J.B., S.B., H.B., N.R., B.A. and T.K.S. Supervision: M.G.N. and K.P. Project administration: K.P. Funding acquisition: M.G.N. and K.P. All authors have read and agreed to the published version of the manuscript.

## Conflicts of Interest

L.A.B.J. and M.G.N. are scientific founders of TTxD and Lemba. The other authors declare no conflicts of interest.

## Supporting information


**Figure S1.** In vitro mevalonate pathway inhibition impairs cytokine production by CD4 and CD8 T cells. A Schematic gating strategy of Vδ2 T cells, CD4 T cells and CD8 T cells in PBMC cultures. Example shown are IPP‐treated (in the presence of IL‐2) PBMCs cultures for 12 days. (B) Cumulative percentage of live cells in PBMC cultures after incubation with indicated inhibitors (Mean ± SEM, *n* = 7); and (C) in the presence or absence of mevalonic acid (Mean ± SEM, *n* = 6). (D) Cumulative frequency of Vδ2 T cells, TNF^+^ and IFN‐γ^+^ Vδ2 T cells incubated with zoledronate and IL‐2 for 14 days, 18 days and 21 days. (Mean ± SEM, *n* = 6). (E) The memory phenotype of Vδ2 T cells incubated with zoledronate and IL‐2 for 12 days. CD45RA^+^CD27^+^: naïve; CD45RA^−^CD27^−^: effector memory; CD45RA^−^CD27^+^: central memory; CD45RA^+^CD27^−^: terminally differentiated. (Mean ± SEM, *n* = 6). (F) Exemplary FACS plot (upper level) and cumulative percentage of purification efficiency of Vδ2 T cells (*n* = 5) (lower level). (G) Cumulative percentage of TNF^+^ and IFN‐γ^+^ Vδ2 T cells in purified Vδ2 T cell cultures incubated with or without zoledronate in the presence of IL‐2 overnight (O.N.), 2 days and 6 days. (Mean ± SEM, *n* = 5). (H) FACS plot representing TNF‐ and IFN‐γ‐ production capacity by Vδ2 T cells in cultures with over 99% purification efficiency. (I) Cumulative percentage of TNF^+^ and IFN‐γ^+^ Vδ2 T cells in PBMC cultures treated as in Figure 1 with atorvastatin (Ator) and fluvastatin (Fluva) (Mean ± SEM, *n* = 7). (J) Cumulative percentage of TNF^+^ and IFN‐γ^+^ Vδ2 T cells expanded by IPP and IL‐2 in PBMC cultures for 12 days and treated with fluvastatin and zoledronate during the cytokine assay for 4 h. (Mean ± SEM, *n* = 6). Cumulative percentage of TNF^+^ and IFN‐γ^+^ (K) CD4 T cells and (L) CD8 T cells in PBMC cultures treated as in Figure 1b (Mean ± SEM, *n* = 8). (B–L) each dot represents one donor (repeated measures one‐way ANOVA followed by Tukey’s multiple comparisons test, *p value < 0.05).
**Figure S2.** In vivo statin treatment results in compromised cytokine production by CD4 and CD8 T cells. (A, B) Flow cytometry analysis of PBMCs isolated from patients with hypercholesterolemia before and after 3‐months of statin treatment (Mean ± SEM, *n* = 13: patients T0; *n* = 10 patients T1; *n* = 14: healthy donors); (Mann–Whitney test: patients and healthy donors; Wilcoxon test: before and after treatment, *p value < 0.05). (C, D) from hyper IgD patients and from healthy individuals (Mean ± SEM, *n* = 5: patients; *n* = 14: healthy donors); (Mann–Whitney test, *p value < 0.05); cumulative percentage of TNF^+^ and IFN‐γ^+^ (A, C) CD4 T cells and (B, D) CD8 T cells. (A–D) Each dot represents one donor.
**Figure S3.** Transcript expressions of TIM3 and LAG3 are differentially altered in Vδ2 T cells upon zoledronate and fluvastatin treatment, respectively. RNA‐seq analysis of Vδ2 T cells isolated from 12‐days‐PBMC cultures treated with IPP, zoledronate, fluvastatin or RPMI alone. Bar plot showing normalised count of differentially expressed transcripts of PD‐1 (*PDCD1*), TIM3 (*HAVCR2*), *CTLA4* and *LAG3*; T‐bet (*TBX21*), *EOMES*, *TOX* and CD39 (*ENTPD1*) upon IPP, fluvastatin or zoledronate treatment in Vδ2 T cell (*n* = 3).
**Figure S4.** Transcript expression is differentially altered in Vδ2 T cells upon mevalonate pathway inhibition. (A, B) RNA‐seq analysis of Vδ2 T cells isolated from 12‐days‐PBMC cultures treated with IPP, zoledronate, fluvastatin or RPMI alone. (A) Venn diagram demonstrating the numbers of upregulated and downregulated transcripts in Vδ2 T cell in inhibitor‐treated conditions vs. RPMI alone. (B) hCoCena Integrated co‐expression network coloured by cluster and heat map showing mean expression of genes in the modules identified by hCoCena analysis for the RPMI, IPP, zol and statin‐treated groups (C) ATAC‐seq analysis of Vδ2 T cells isolated from 12‐days‐PBMC cultures treated with IPP, zoledronate, fluvastatin or RPMI alone (*n* = 3). Snapshot from the IGV browser of ATAC‐seq signals around the *IFNG* (upper) and *TNF* (lower) loci (*n* = 2). (D) Cumulative percentage of glycolytic and fatty acid oxidation (FAO) & amino acid oxidation (AAO) capacities by Vδ2 T cells incubated with or without IPP, statin, zoledronate or 6‐fluoromevalonate in the presence of IL‐2 for 12 days (Mean ± SEM, *n* = 4) (repeated measures one‐way ANOVA followed by Tukey’s multiple comparisons test, *p value < 0.05).
**Figure S5.** Mevalonate pathway inhibition does not affect cholesterol levels in Vδ2 T cells. Flow cytometry analysis of PBMC cultures treated as in Figure 5A. (A) Representative FACS histogram showing MFI of filipin in Vδ2 T cells after incubation with methyl‐β‐cyclodextrin in PBMC cultures overnight. (B) Bar plot showing MFI of filipin in Vδ2 T cells upon mevalonate pathway inhibition in PBMC cultures (Mean ± SEM, *n* = 4; methyl‐β‐cyclodextrin: *n* = 2) (mixed‐effects analysis followed by Tukey’s multiple comparisons test, *p value < 0.05). (C) Cumulative percentage of TNF^+^ and IFN‐γ^+^ Vδ2 T cells in PBMC cultures treated as in Figure 6A with the following GGTIs at indicated concentrations: GGTI 286 and GGTI 298 (Mean ± SEM, *n* = 5). Cumulative percentage of TNF^+^ and IFN‐γ^+^ (D) CD4 T cells and (E) CD8 T cells in PBMC cultures after incubation in indicated conditions (Mean ± SEM, *n* = 5) (repeated measures one‐way ANOVA followed by Tukey’s multiple comparisons test, *p value < 0.05).
**Figure S6.** Phosphosites downstream of small G proteins show reduced phosphorylation levels in both statin‐ and zoledronate‐treated Vδ2 T cells. (A) Heatmap showing differentially affected phosphosites in IPP‐, zoledronate‐ and fluvastatin‐ vs. RPMI‐treated Vδ2 T cells. (B) Venn plot showing number of kinases with up‐ and down‐regulated activity in IPP‐, zoledronate‐ and fluvastatin‐ vs. RPMI‐treated Vδ2 T cells.
**Figure S7.** Mevalonate metabolism is important for cytotoxic properties of CD8 T cells in vitro. A Cumulative percentage of granzyme B^+^ and perforin^+^ CD8 T cells in PBMC cultures after incubation with indicated inhibitors, assessed as in Figure 7A (Mean ± SEM, *n* = 5) (repeated measures one‐way ANOVA followed by Tukey’s multiple comparisons test, *p value < 0.05). (B) Cumulative percentage of granzyme B^+^ and perforin^+^ CD8 T cells in patients with hypercholesterolemia before and after 3‐months of statin treatment (Mean ± SEM, *n* = 10: patients T0, T1; *n* = 10: healthy donors) (Mann–Whitney test: patients and healthy donors; Wilcoxon test: before and after treatment, *p value * < 0.05).

**Table S1.** General characteristics of statin trial participants.

**Table S2.** General characteristics of Hyper IgD trial participants.

## Data Availability

RNA‐seq and ATAC‐seq data have been deposited at EGA and are publicly available as of the date of publication. The accession number is EGAD00001011322.
