# Supplementary material for: Human γδ T Cell Function Is Impaired Upon Mevalonate Pathway Inhibition
Source: Immunology. 2025 Apr 22;175(3):300–22. doi: 10.1111/imm.13931 (PMC12130672; doi:10.1111/imm.13931)
Supplement: Supplementary file 1 — Figure S1. In vitro mevalonate pathway inhibition impairs cytokine production by CD4 and CD8 T cells. A Schematic gating strategy of Vδ2 T cells, CD4 T cells and CD8 T cells in PBMC cultures. Example shown are IPP‐treated (in the presence of IL‐2) PBMCs cultures for 12 days. (B) Cumulative percentage of live cells in PBMC cultures after incubation with indicated inhibitors (Mean ± SEM, n = 7); and (C) in the presence or absence of mevalonic acid (Mean ± SEM, n = 6). (D) Cumulative frequency of Vδ2 T cells, TNF+ and IFN‐γ+ Vδ2 T cells incubated with zoledronate and IL‐2 for 14 days, 18 days and 21 days. (Mean ± SEM, n = 6). (E) The memory phenotype of Vδ2 T cells incubated with zoledronate and IL‐2 for 12 days. CD45RA+CD27+: naïve; CD45RA−CD27−: effector memory; CD45RA−CD27+: central memory; CD45RA+CD27−: terminally differentiated. (Mean ± SEM, n = 6). (F) Exemplary FACS plot (upper level) and cumulative percentage of purification efficiency of Vδ2 T cells (n = 5) (lower level). (G) Cumulative percentage of TNF+ and IFN‐γ+ Vδ2 T cells in purified Vδ2 T cell cultures incubated with or without zoledronate in the presence of IL‐2 overnight (O.N.), 2 days and 6 days. (Mean ± SEM, n = 5). (H) FACS plot representing TNF‐ and IFN‐γ‐ production capacity by Vδ2 T cells in cultures with over 99% purification efficiency. (I) Cumulative percentage of TNF+ and IFN‐γ+ Vδ2 T cells in PBMC cultures treated as in Figure 1 with atorvastatin (Ator) and fluvastatin (Fluva) (Mean ± SEM, n = 7). (J) Cumulative percentage of TNF+ and IFN‐γ+ Vδ2 T cells expanded by IPP and IL‐2 in PBMC cultures for 12 days and treated with fluvastatin and zoledronate during the cytokine assay for 4 h. (Mean ± SEM, n = 6). Cumulative percentage of TNF+ and IFN‐γ+ (K) CD4 T cells and (L) CD8 T cells in PBMC cultures treated as in Figure 1b (Mean ± SEM, n = 8). (B–L) each dot represents one donor (repeated measures one‐way ANOVA followed by Tukey’s multiple comparisons test, *p value < 0.05). Figure S2. In viv [file IMM-175-300-s001.pdf]

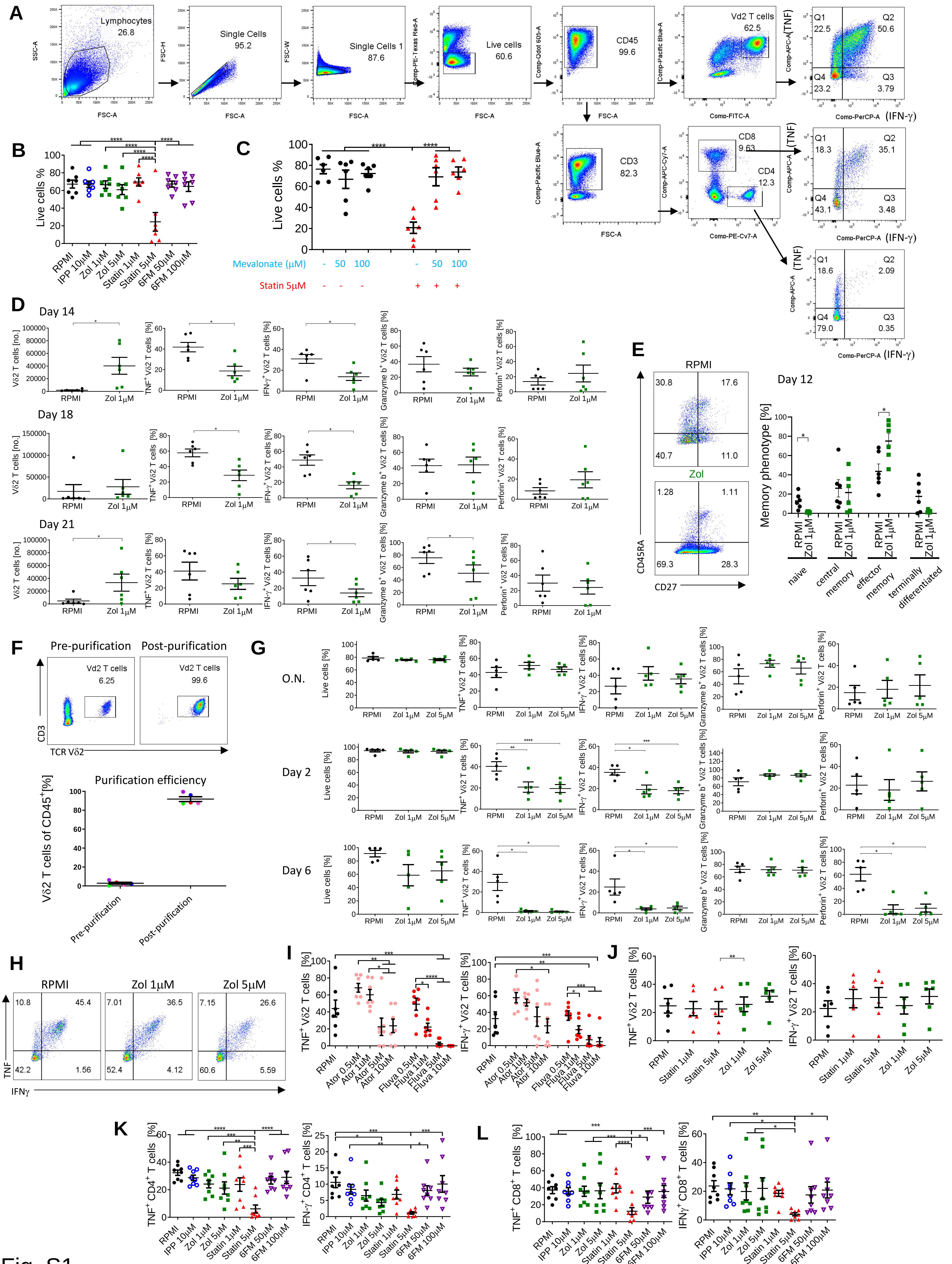

Fig. S1

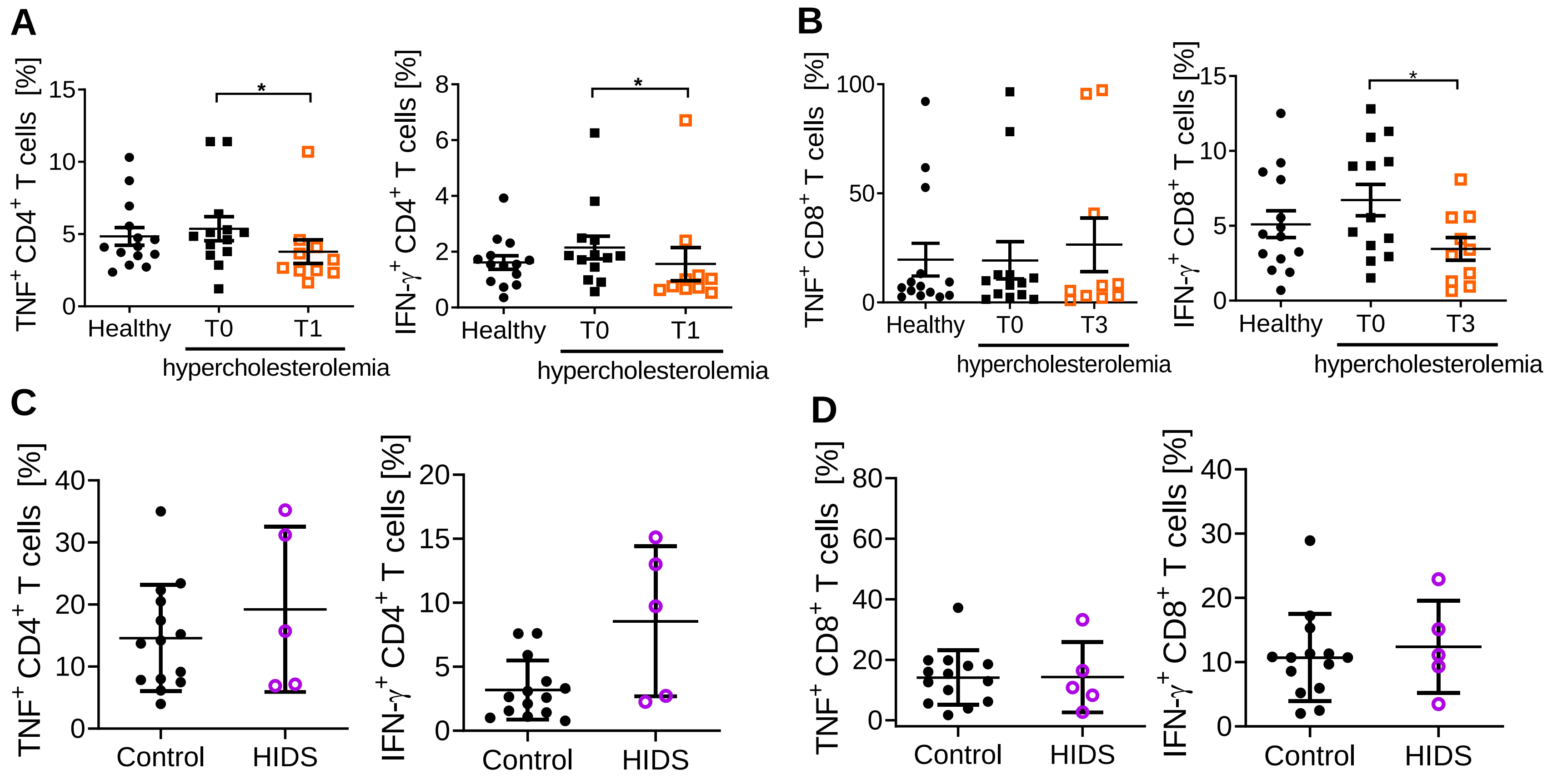
A
TNF+ CD4+ T cells [%]
Healthy
T0
T1
hypercholesterolemia
IFN-γ+ CD4+ T cells [%]
B
TNF+ CD8+ T cells [%]
T3
IFN-γ+ CD8+ T cells [%]
C
Control
HIDS
D

Fig. S2

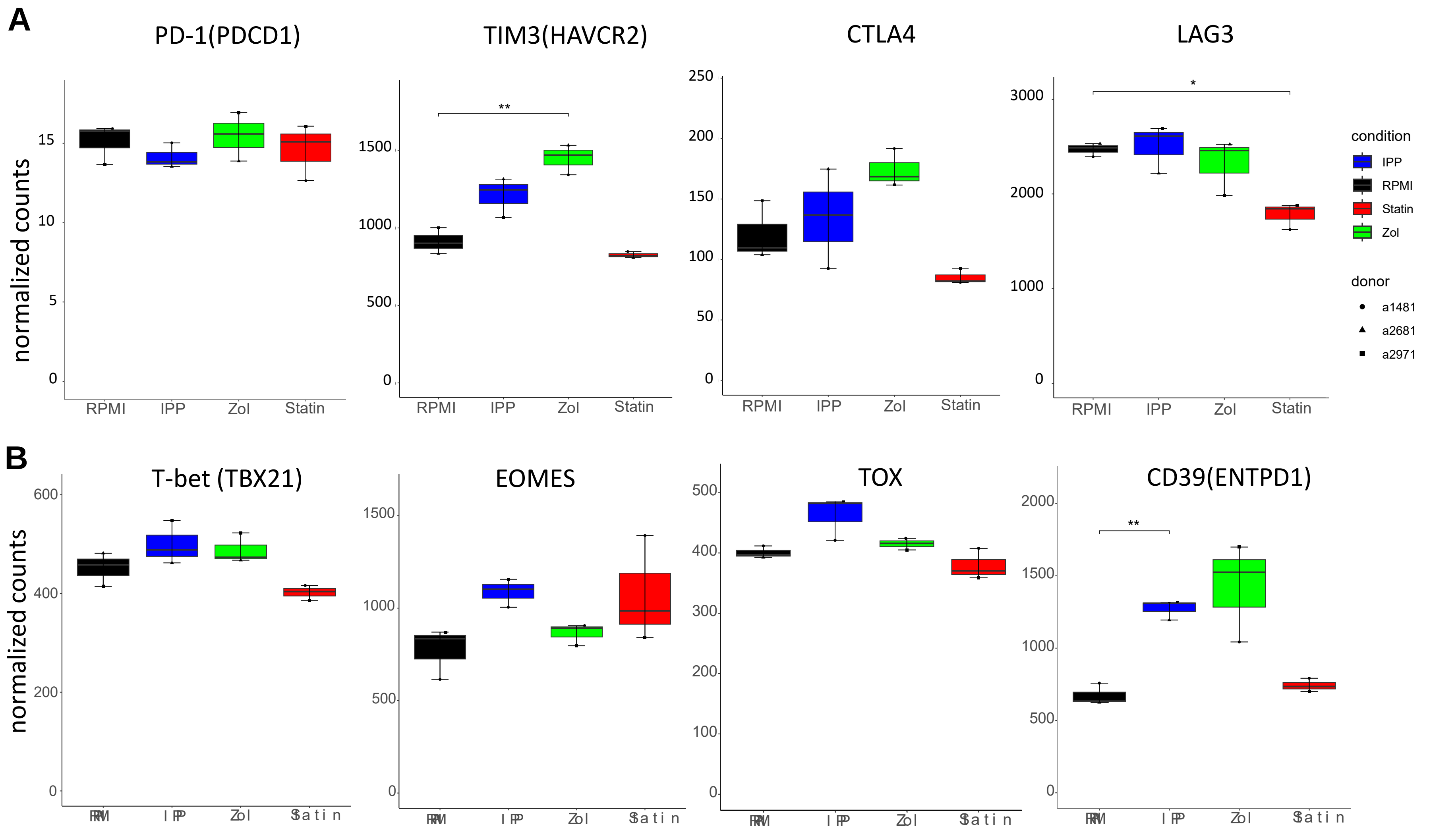
A
PD-1(PDCD1)
TIM3(HAVCR2)
CTLA4
LAG3
normalized counts
RPMI
IPP
Zol
Statin
condition
donor
a1481
a2681
a2971
B
T-bet (TBX21)
EOMES
TOX
CD39(ENTPD1)

Fig. S3

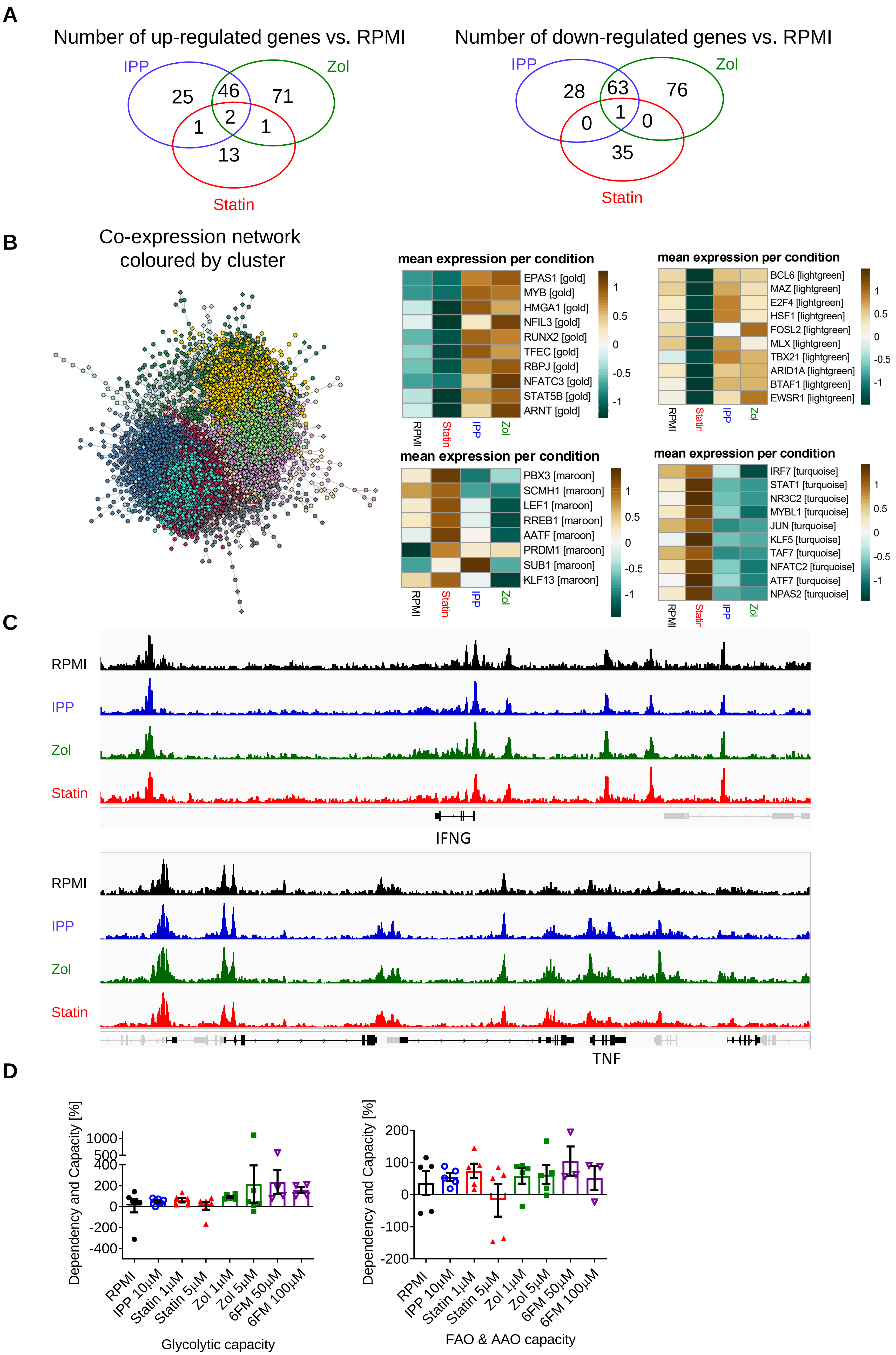
A
Number of up-regulated genes vs. RPMI
IPP
Zol
Statin
25
46
71
2
1
1
13
Number of down-regulated genes vs. RPMI
IPP
Zol
Statin
28
63
76
1
0
0
35
B
Co-expression network
coloured by cluster
mean expression per condition
EPAS1 [gold]
MYB [gold]
HMGA1 [gold]
NFIL3 [gold]
RUNX2 [gold]
TFEC [gold]
RBPJ [gold]
NFATC3 [gold]
STAT5B [gold]
ARNT [gold]
mean expression per condition
BCL6 [lightgreen]
MAZ [lightgreen]
E2F4 [lightgreen]
HSF1 [lightgreen]
FOSL2 [lightgreen]
MLX [lightgreen]
TBX21 [lightgreen]
ARID1A [lightgreen]
BTAF1 [lightgreen]
EWSR1 [lightgreen]
mean expression per condition
PBX3 [maroon]
SCMH1 [maroon]
LEF1 [maroon]
RREB1 [maroon]
AATF [maroon]
PRDM1 [maroon]
SUB1 [maroon]
KLF13 [maroon]
mean expression per condition
IRF7 [turquoise]
STAT1 [turquoise]
NR3C2 [turquoise]
MYBL1 [turquoise]
JUN [turquoise]
KLF5 [turquoise]
TAF7 [turquoise]
NFATC2 [turquoise]
ATF7 [turquoise]
NPAS2 [turquoise]
1
0.5
0
-0.5
-1
RPMI
Statin
IPP
Zol
C
RPMI
IPP
Zol
Statin
IFNG
RPMI
IPP
Zol
Statin
TNF
D
Dependency and Capacity [%]
1000
500
400
200
0
-200
-400
RPMI
IPP 10µM
Statin 1µM
Statin 5µM
Zol 1µM
Zol 5µM
6FM 50µM
6FM 100µM
Glycolytic capacity
Dependency and Capacity [%]
200
100
0
-100
-200
RPMI
IPP 10µM
Statin 1µM
Statin 5µM
Zol 1µM
Zol 5µM
6FM 50µM
6FM 100µM
FAO & AAO capacity

Fig. S4

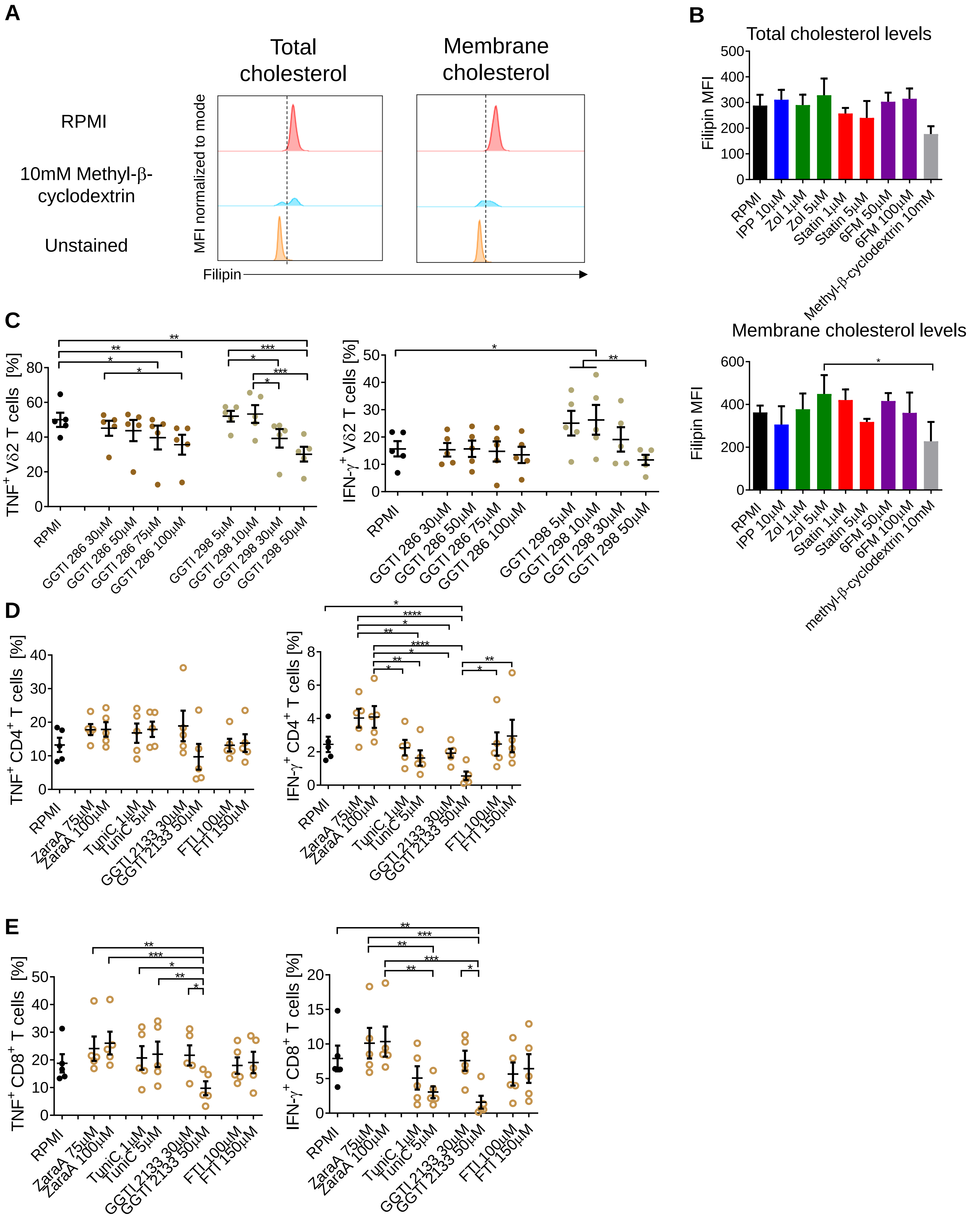
A
Total cholesterol
Membrane cholesterol
RPMI
10mM Methyl-β-cyclodextrin
Unstained
MFI normalized to mode
Filipin
B
Total cholesterol levels
Filipin MFI
RPMI
IPP 10µM
Zol 1µM
Zol 5µM
Statin 1µM
Statin 5µM
6FM 50µM
6FM 100µM
Methyl-β-cyclodextrin 10mM
Membrane cholesterol levels
methyl-β-cyclodextrin 10mM
C
TNF+ Vδ2 T cells [%]
IFN-γ+ Vδ2 T cells [%]
GGTI 286 30µM
GGTI 286 50µM
GGTI 286 75µM
GGTI 286 100µM
GGTI 298 5µM
GGTI 298 10µM
GGTI 298 30µM
GGTI 298 50µM
D
TNF+ CD4+ T cells [%]
IFN-γ+ CD4+ T cells [%]
ZaraA 75µM
ZaraA 100µM
TuniC 1µM
TuniC 5µM
GGTI 2133 30µM
GGTI 2133 50µM
FTI 100µM
FTI 150µM
E
TNF+ CD8+ T cells [%]
IFN-γ+ CD8+ T cells [%]

Fig. S5

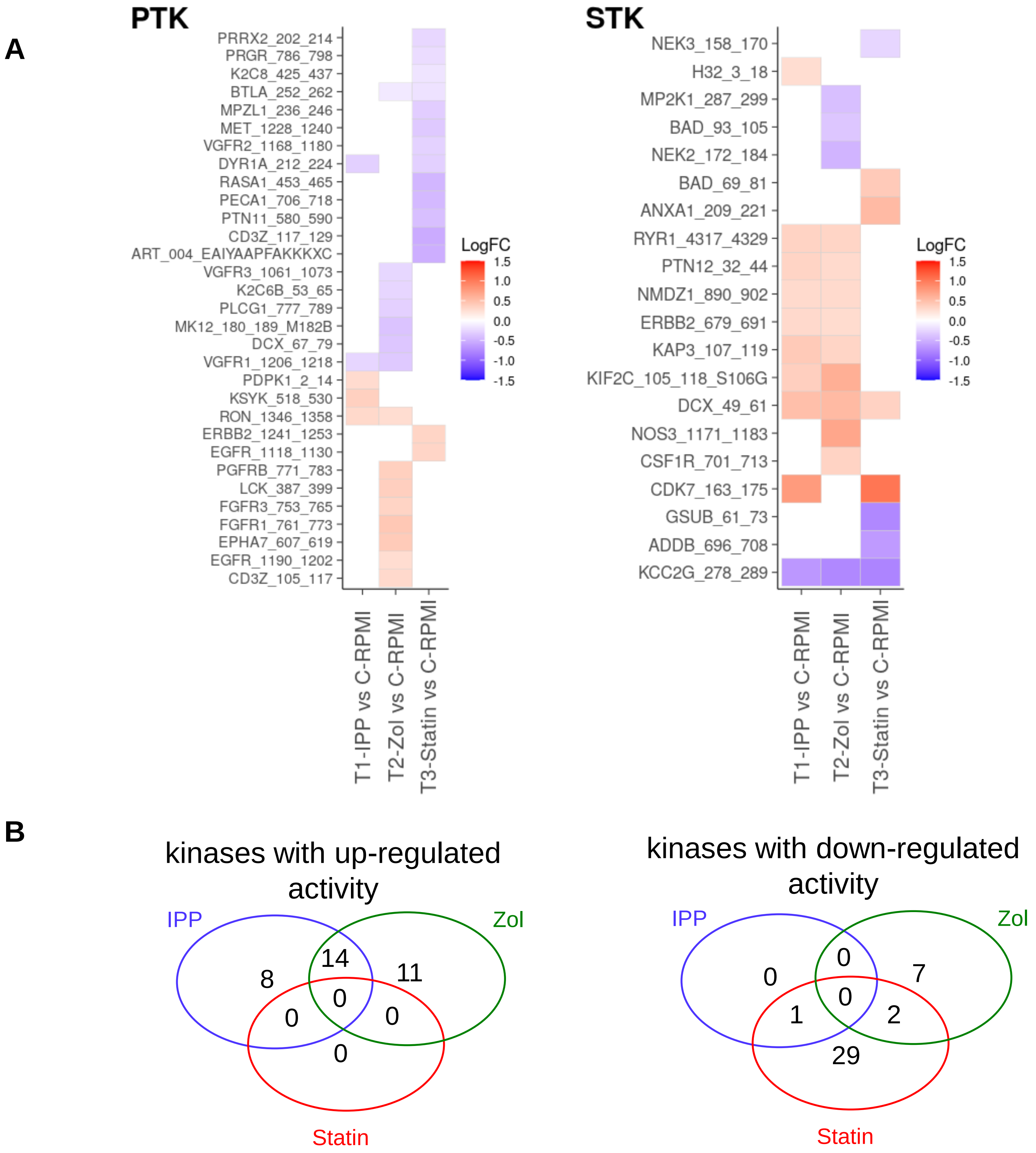
A
PTK
PRRX2_202_214
PRGR_786_798
K2C8_425_437
BTLA_252_262
MPZL1_236_246
MET_1228_1240
VGFR2_1168_1180
DYR1A_212_224
RASA1_453_465
PECA1_706_718
PTN11_580_590
CD3Z_117_129
ART_004_EAIYAAPFAKKKXC
VGFR3_1061_1073
K2C6B_53_65
PLCG1_777_789
MK12_180_189_M182B
DCX_67_79
VGFR1_1206_1218
PDPK1_2_14
KSYK_518_530
RON_1346_1358
ERBB2_1241_1253
EGFR_1118_1130
PGFRB_771_783
LCK_387_399
FGFR3_753_765
FGFR1_761_773
EPHA7_607_619
EGFR_1190_1202
CD3Z_105_117
T1-IPP vs C-RPMI
T2-Zol vs C-RPMI
T3-Statin vs C-RPMI
LogFC
1.5
1.0
0.5
0.0
-0.5
-1.0
-1.5
STK
NEK3_158_170
H32_3_18
MP2K1_287_299
BAD_93_105
NEK2_172_184
BAD_69_81
ANXA1_209_221
RYR1_4317_4329
PTN12_32_44
NMDZ1_890_902
ERBB2_679_691
KAP3_107_119
KIF2C_105_118_S106G
DCX_49_61
NOS3_1171_1183
CSF1R_701_713
CDK7_163_175
GSUB_61_73
ADDB_696_708
KCC2G_278_289
T1-IPP vs C-RPMI
T2-Zol vs C-RPMI
T3-Statin vs C-RPMI
LogFC
1.5
1.0
0.5
0.0
-0.5
-1.0
-1.5
B
kinases with up-regulated activity
IPP
Zol
Statin
8
14
11
0
0
0
0
kinases with down-regulated activity
IPP
Zol
Statin
0
0
7
1
0
2
29

Fig. S6

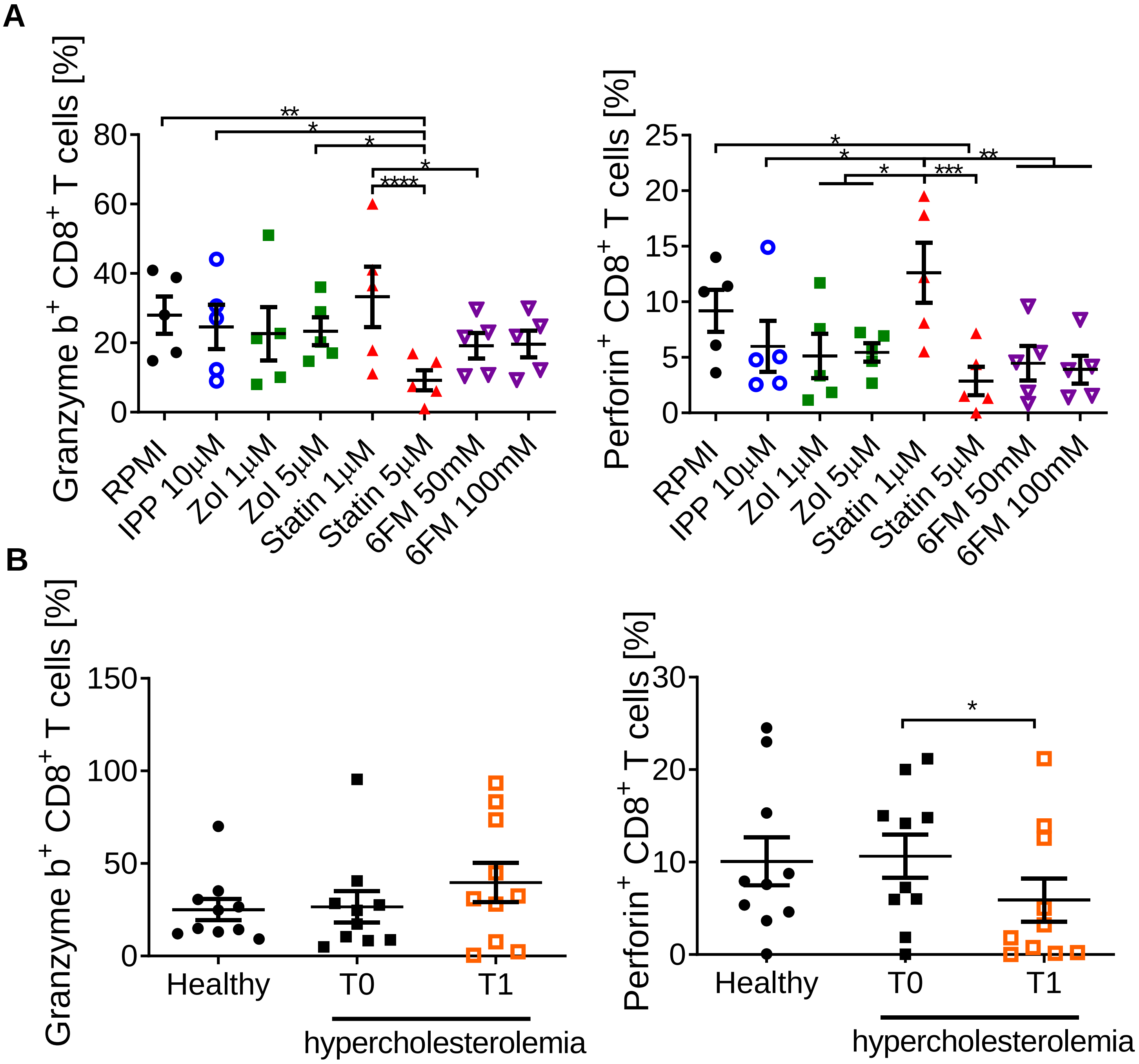
A
Granzyme b+ CD8+ T cells [%]
80
60
40
20
0
**
*
*
*
****
RPMI
IPP 10µM
Zol 1µM
Zol 5µM
Statin 1µM
Statin 5µM
6FM 50mM
6FM 100mM
Perforin+ CD8+ T cells [%]
25
20
15
10
5
0
*
*
*
***
**
B
Granzyme b+ CD8+ T cells [%]
150
100
50
0
Healthy
T0
T1
hypercholesterolemia
Perforin+ CD8+ T cells [%]
30
20
10
0
*

Fig. S7
